# The genetic architecture of colonization resistance in *Brachypodium distachyon* to non-adapted stripe rust (*Puccinia striiformis*) isolates

**DOI:** 10.1371/journal.pgen.1007637

**Published:** 2018-09-28

**Authors:** Jan Bettgenhaeuser, Matthew Gardiner, Rebecca Spanner, Phon Green, Inmaculada Hernández-Pinzón, Amelia Hubbard, Michael Ayliffe, Matthew J. Moscou

**Affiliations:** 1 The Sainsbury Laboratory, Norwich, United Kingdom; 2 National Institute of Agricultural Botany, Cambridge, United Kingdom; 3 Commonwealth Scientific and Industrial Research Organisation, Agriculture and Food, Canberra, Australian Capital Territory, Australia; 4 School of Biological Sciences, University of East Anglia, Norwich, United Kingdom; Danforth Center, UNITED STATES

## Abstract

Multilayered defense responses ensure that plants are hosts to only a few adapted pathogens in the environment. The host range of a plant pathogen depends on its ability to fully overcome plant defense barriers, with failure at any single step sufficient to prevent life cycle completion of the pathogen. *Puccinia striiformis*, the causal agent of stripe rust (=yellow rust), is an agronomically important obligate biotrophic fungal pathogen of wheat and barley. It is generally unable to complete its life cycle on the non-adapted wild grass species *Brachypodium distachyon*, but natural variation exists for the degree of hyphal colonization by *Puccinia striiformis*. Using three *B*. *distachyon* mapping populations, we identified genetic loci conferring colonization resistance to wheat-adapted and barley-adapted isolates of *P*. *striiformis*. We observed a genetic architecture composed of two major effect QTLs (*Yrr1* and *Yrr3*) restricting the colonization of *P*. *striiformis*. Isolate specificity was observed for *Yrr1*, whereas *Yrr3* was effective against all tested *P*. *striiformis* isolates. Plant immune receptors of the nucleotide binding, leucine-rich repeat (NB-LRR) encoding gene family are present at the *Yrr3* locus, whereas genes of this family were not identified at the *Yrr1* locus. While it has been proposed that resistance to adapted and non-adapted pathogens are inherently different, the observation of (1) a simple genetic architecture of colonization resistance, (2) isolate specificity of major and minor effect QTLs, and (3) NB-LRR encoding genes at the *Yrr3* locus suggest that factors associated with resistance to adapted pathogens are also critical for non-adapted pathogens.

## Author summary

Plants are constantly exposed to a multitude of potential pathogens but remain immune to most of these due to a multilayered immune system. Pathogens have specialized by adapting to certain host plants and their defense barriers. Most of our understanding of plant-pathogen interactions stems from these highly specialized interactions, because they are characterized by qualitative interactions (resistant or susceptible). It has generally been assumed that the genetic and molecular basis of resistance to non-adapted pathogens is fundamentally different, as either no variation exists in a species (complete immunity) or variation encompasses only early pathogen invasion (colonization), but not full susceptibility. We have studied the interaction between the agronomically important fungal stripe rust pathogen (*Puccinia striiformis*) of wheat and barley with the wild grass species *Brachypodium distachyon*. Rust infections consist of two stages: colonization of plant tissues followed by a reproductive phase. We identified natural variation for the degree of *P*. *striiformis* colonization in different *B*. *distachyon* accessions and dissected the genetic architecture controlling resistance at this infection stage. QTLs conferring resistance possessed several characteristics similar to adapted host systems, indicating that resistance to adapted and non-adapted pathogens are not intrinsically different.

## Introduction

An integral characteristic of plant-pathogen interactions are the several events that lead to infection of a plant by a pathogen. To successfully complete its life cycle, that is to colonize a plant and reproduce, a plant pathogen needs to overcome several preformed and inducible barriers [1]. Successful life cycle completion relies on compatibility at all of these stages and incompatibility at only one stage prevents pathogen reproduction. Because of this, a plant is generally resistant to the vast majority of potential pathogens in the environment and only susceptible to a small number of adapted pathogens [2]. Additionally, colonization of new plant species by plant pathogens is considered a rare event [3], as any new pathogen would have to overcome all defense barriers employed by the new plant species.

Prior to the arrival of a pathogen, plants have several preformed barriers that will limit infection. Examples include the leaf surface composition, which can prevent germination and differentiation of the plant pathogen, or antimicrobial molecules, such as avenacins of oat that can prevent pathogen growth in leaf tissue [1, 4, 5]. Once a plant pathogen evades preformed barriers, recognition of the attempted infection may occur and initiate the deployment of inducible barriers [1]. Examples of inducible barriers include the three *PENETRATION* (*PEN*) genes of *Arabidopsis thaliana*, which regulate the structural rearrangements necessary for the formation of papillae, localized reinforcements of the cell wall that prevent pathogen colonization [2, 6–9]. *PEN* gene expression is induced upon flagellin perception, a bacterial pathogen-associated molecular pattern (PAMP), by the membrane-localized plant immune receptor FLS2, a receptor-like kinase [7, 10]. Recognition at the membrane can be overcome by plant pathogens through the secretion of effector molecules into plant cells [11, 12]. In turn, plants have evolved nucleotide binding, leucine-rich repeat (NB-LRR) proteins that recognize effector molecules or effector modifications of plant proteins. By initiating localized cell death, also called hypersensitive response, this recognition forms a further defense layer [13–15]. These late stages of the plant-pathogen interaction are conceptualized as PAMP-triggered immunity (PTI) and effector-triggered immunity (ETI). ETI can be suppressed by additional pathogen effectors, prompting an evolutionary arms race between plant and pathogen [13]. The interactions between NB-LRRs and effectors are commonly genetically observed as a gene-for-gene interaction between the host plant and an adapted pathogen [13, 16].

*Puccinia striiformis*, causal agent of stripe or yellow rust, is an agronomically important obligate biotrophic fungal pathogen of wheat, barley, and other domesticated crops, as well as many non-domesticated grasses [17–19]. Following stomatal penetration, the first stage of *P*. *striiformis* development involves hyphal differentiation and colonization of host leaf tissue through the formation of haustoria for nutrient acquisition and effector secretion [18]. After substantial colonization of a compatible host, *P*. *striiformis* transitions to a reproductive stage through the development of urediniospores, which completes the asexual reproductive lifecycle [18, 20]. Sexual reproduction involves additional spore stages on the alternative host, *Berberis spp*. [18, 21]. *P*. *striiformis* isolates adapted to certain host genera are differentiated as *formae speciales*, including *P*. *striiformis* f. sp. *tritici* with wheat as the main host (wheat stripe rust, *Pst*) and *P*. *striiformis* f. sp. *hordei* with barley as the main host (barley stripe rust, *Psh*) [22]. However, this classification is complicated by the existence of *formae speciales* with overlapping host ranges. For example, a *P*. *striiformis* race emerged on triticale in Denmark and Sweden in 2008 and 2009, which also infected spring wheat, barley, and rye [18, 23].

Straib [24] investigated the host range of *Pst* and *Psh* isolates on a panel of 227 mainly non-domesticated grass species and observed chlorotic or necrotic flecks as well as pustule formation in some genera. The panel included an accession of the diploid monocot model *Brachypodium distachyon*, which was completely immune to the isolates studied. Draper *et al*. [25] identified *B*. *distachyon* accessions that produced disease symptoms in the form of “brown flecking” upon *Pst* and *Psh* inoculation. These observations were confirmed by Barbieri *et al*. [26], who described “large dark flecks” on some *B*. *distachyon* accessions in response to infection with *Pst* and *Psh* isolates. A comprehensive analysis of *B*. *distachyon–Pst* interactions linked these macroscopic flecks with hyphal colonization [27], which led to the application of a robust and quantitative phenotyping assay to a diversity set of *Brachypodium* spp. accessions inoculated with two UK *Pst* isolates [28]. A strong correlation between macroscopic leaf browning and hyphal colonization was observed across 210 *Brachypodium* spp. accessions.

Although host jumps are considered rare events, pathogens are often able to infect or colonize plants other than their adapted host with varying degrees of success [29]. As exemplified by the interaction between *P*. *striiformis* and *B*. *distachyon*, a range of phenotypes are observed that are difficult to assign to a host (full compatibility) or nonhost (full incompatibility) state. Therefore, the status of species can be described by the range of colonization and life cycle completion by the pathogen [30]. This classification is based on the diversity observed at the species level for both plant and rust. In the case of intermediate nonhost species, no accession would support life cycle completion by different rust isolates, but some accessions would allow a degree of colonization. The above-mentioned studies established *B*. *distachyon* as an intermediate nonhost of *Pst* and *Psh*. In contrast, rice is considered a nonhost of rusts, as no accessions have been identified that allow extensive colonization or further disease progression [31–33].

*P*. *brachypodii* is an adapted rust pathogen of *B*. *distachyon* and the related *B*. *sylvaticum* [26, 34]. Therefore, unlike for rice, fully compatible interactions exist between *B*. *distachyon* and a rust pathogen. Consequently, the *B*. *distachyon–P*. *striiformis* interaction provides a unique system to study the genetic architecture underlying defense responses against non-adapted rust pathogens. Using three differential *B*. *distachyon* mapping populations and a quantitative microscopic assay, we investigated colonization resistance to *P*. *striiformis*. We found that the ability of four diverse *P*. *striiformis* isolates (three *Pst* and one *Psh*) to colonize *B*. *distachyon* leaves is governed by a simple genetic architecture, with resistance largely provided by two major effect QTLs (*Yrr1* and *Yrr3*). *Yrr3* is functional against all *Pst* and *Psh* isolates tested, while *Yrr1* mediates resistance to the *Pst* isolates only. These findings show that although plant defense responses to non-adapted pathogens are multilayered, the genetic basis of individual layers of resistance resemble the complexity of host resistance.

## Results

The quantitative nature of phenotypes observed in the interactions between plants and non-adapted pathogens has provided an obstacle to studying the genetic and molecular basis of these intermediate interactions [29]. Previous studies described natural variation for hyphal colonization and leaf browning in response to *P*. *striiformis* infection among diverse *B*. *distachyon* accessions [27, 28]. Our investigation focused on dissecting the relationship of phenotype and genotype to understand the architecture underlying colonization resistance to diverse non-adapted *P*. *striiformis* isolates in *B*. *distachyon*.

### Leaf browning and *Pst* hyphal colonization are strongly correlated in segregating *B*. *distachyon* mapping populations

**Fig 1:**
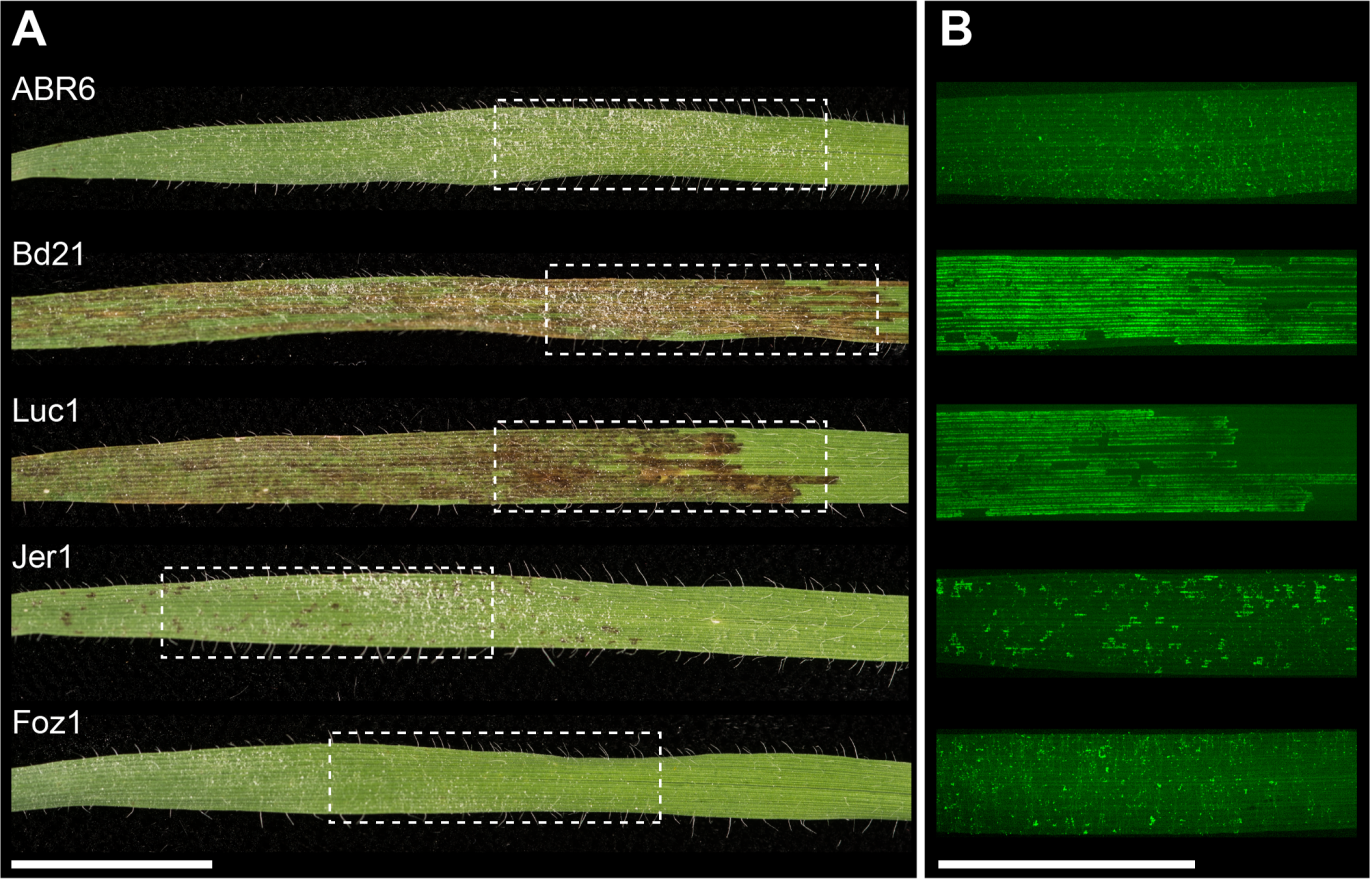
*Puccinia striiformis* f. sp. *tritici* (*Pst*; wheat stripe rust) infection symptoms on several *Brachypodium distachyon* accessions. (A) Leaf browning 14 days after inoculation with *Pst* isolate 08/21. (B) Micrograph of the same leaves cleared and stained with a chitin-binding fluorophore (WGA-FITC) to visualize hyphal growth. Boxed leaf area in (A) corresponds approximately to leaf area in (B). The bars are equal to 10 mm.

In the *B*. *distachyon*–*Pst* interaction, macroscopic infection symptoms manifest as leaf browning (Fig 1). In a survey of 210 *Brachypodium* spp. accessions, strong correlation was found between macroscopic leaf browning (Fig 1A) and hyphal growth (percent colonization, pCOL; Fig 1B) of the *Pst* isolate 08/21 [28]. While leaf browning and hyphal colonization are correlated traits in diverse germplasm, it is unknown whether a shared genetic architecture controls these phenotypes. We hypothesized that leaf browning and pCOL have a shared genetic architecture. To test this, we assessed these phenotypes in the interaction of *Pst* isolate 08/21 and three segregating *B*. *distachyon* populations.

The ABR6 x Bd21 F_4:5_ population was derived from a cross between accessions collected from geographically distinct regions, i.e. Spain (accession ABR6) and Iraq (reference accession Bd21) [35]. These two accessions differ substantially at the genomic level [35, 36]. ABR6 does not develop any macroscopic symptoms following *Pst* infection, whereas Bd21 displays leaf browning and allows hyphal growth (Fig 1). Leaf browning and pCOL phenotypes in the ABR6 x Bd21 F_4:5_ population were not normally distributed and heavily skewed towards resistance (S1A and S1B Fig). The segregation pattern for pCOL phenotypes displayed a broader distribution than leaf browning and transgressive segregation for more colonization than Bd21 was observed. Leaf browning and pCOL showed strong correlation (ρ = 0.85; S1C Fig).

Upon infection with *Pst*, *B*. *distachyon* accessions collected in the western Mediterranean (predominantly Spain) displayed greater phenotypic diversity than accessions derived from the eastern Mediterranean (Turkey to Iraq), ranging from large macroscopic lesions to complete microscopic immunity [28]. Three Spanish accessions were selected to generate F_2_ populations: Foz1 does not develop any infection symptoms and Jer1 only displays very small browning and colonization sites, whereas Luc1 leaves become heavily colonized after infection (Fig 1). For the Foz1 x Luc1 and Luc1 x Jer1 populations, 188 F_2_ individuals were evaluated for leaf browning at 14 days post inoculation (dpi) and for both leaf browning and pCOL at 23 dpi. Similar to observations on the ABR6 x Bd21 F_4:5_ population, the leaf browning and pCOL phenotypes were not normally distributed. All three phenotyping results for the Foz1 x Luc1 F_2_ population were skewed towards resistance (S2A–S2C Fig), as were the phenotyping results for the Luc1 x Jer1 F_2_ population at 14 dpi (S2E Fig). Interestingly, at 23 dpi leaf browning phenotypes were distributed uniformly and the pCOL phenotypes were almost normally distributed in the Luc1 x Jer1 F_2_ population (S2F and S2G Fig). At 23 dpi the leaf browning and pCOL phenotypes were correlated with correlation coefficients of 0.86 and 0.76 for the Foz1 x Luc1 and Luc1 x Jer1 F_2_ populations, respectively (S2D and S2H Fig). Transgressive segregation towards increased colonization was observed in the Foz1 x Luc1 F_2_ population and towards increased resistance and colonization in the Luc1 x Jer1 F_2_ population. Strong correlation of leaf browning and pCOL in segregating populations indicates that these macroscopic and microscopic phenotypes share a similar genetic architecture. This is further supported by the overlapping physical localization of these phenotypes (Fig 1), suggesting that fungal development contributes to the macroscopic physiological response of infected *B*. *distachyon* leaves.

### Two major QTLs underlie resistance to *Pst* isolate 08/21 in three *B*. *distachyon* mapping populations

To explore the complexity of the genetic architecture of this interaction, SNP-based genetic maps were created for the Foz1 x Luc1 and Luc1 x Jer1 F_2_ populations. A genetic map was previously developed for the ABR6 x Bd21 F_4:5_ population [35]. The Foz1 x Luc1 genetic map is based on 179 genotyped F_2_ lines, contains 101 non-redundant markers, and has a cumulative size of 1,430 cM (S3 Fig). The Luc1 x Jer1 genetic map is based on 188 genotyped F_2_ lines, contains 107 markers, and has a cumulative size of 1,446 cM (S4 Fig). Both genetic maps have five linkage groups, corresponding to the five chromosomes of *B*. *distachyon*. The quality and integrity of these genetic maps were confirmed by assessing two-way recombination fraction plots for all markers (S5 Fig) and by analyzing all chromosomes for segregation distortion and missing data (S6 Fig).

Linkage analyses using composite interval mapping were performed on all three mapping populations in order to determine the genetic architecture underlying resistance to the UK *Pst* isolate 08/21. For the ABR6 x Bd21 F_4:5_ population, linkage analyses were performed with phenotypic scores from averaged and individual replicates. Linkage analyses were performed for 179 and 188 genotyped F_2_ lines in the Foz1 x Luc1 and Luc1 x Jer1 F_2_ populations, and further validated with 95 F_2:3_ derived families from the Luc1 x Jer1 F_2_ population. Both leaf browning and pCOL were assessed for all three populations. Loci that significantly contributed to resistance were designated *Yrr* (*Yellow rust resistance*), based on the naming convention for resistance loci in *B*. *distachyon* [37].

**Fig 2:**
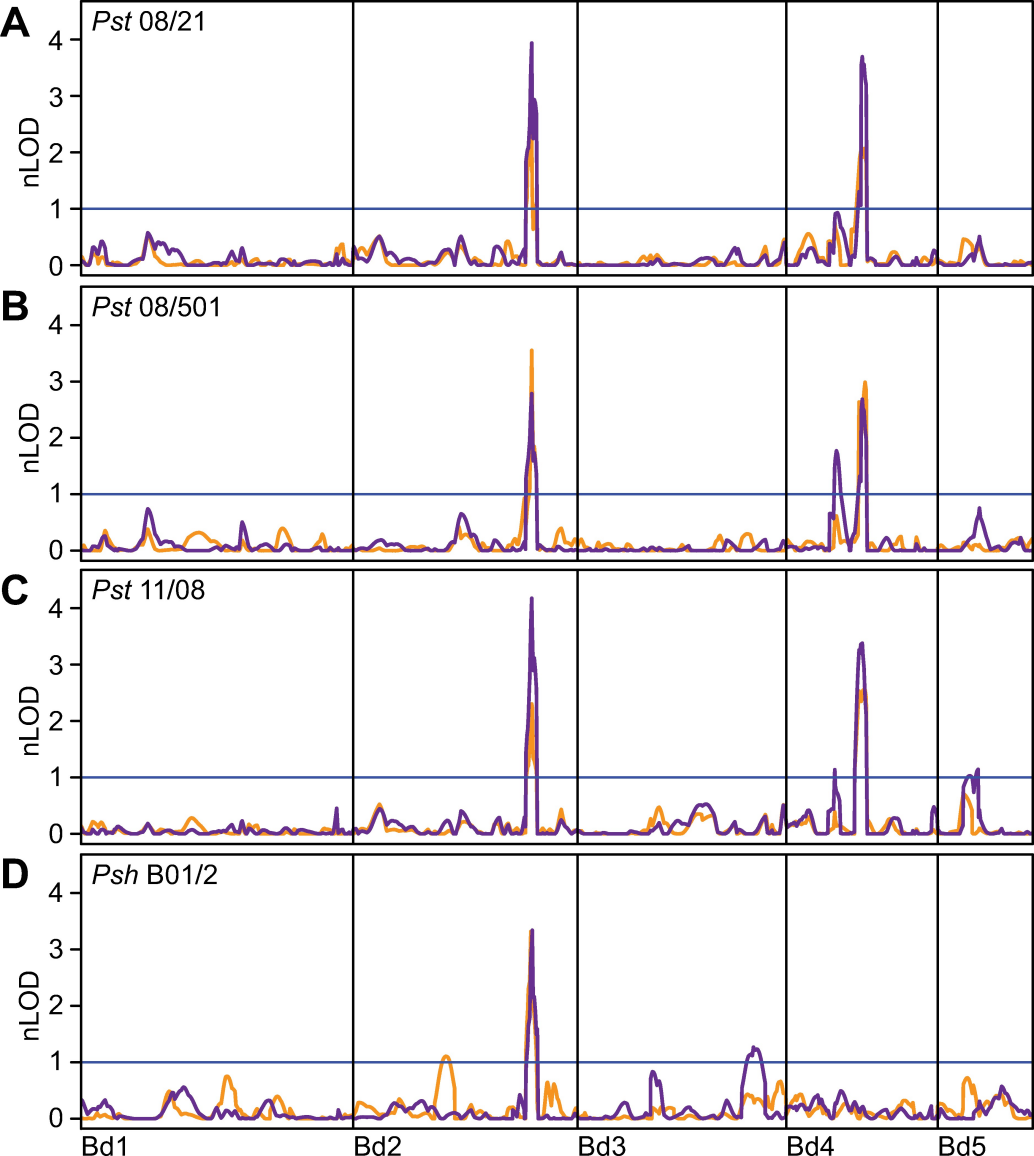
Two major effect loci govern *P*. *striiformis* resistance in the ABR6 x Bd21 F_4:5_ population. Composite interval mapping using averaged phenotypes of F_4:5_ families scored 14 days post inoculation with *P*. *striiformis* f. sp. *tritici* (*Pst*) isolates 08/21 (A), 08/501 (B), and 11/08 (C), and *P*. *striiformis* f. sp. *hordei* (*Psh*) isolate B01/2 (D). Leaf browning (orange) and pCOL (purple) were averaged across replicates before performing linkage analyses using an additive model (H_0_:H_1_). Results were plotted based on normalized permutation thresholds (nLOD), using the threshold of statistical significance based on 1,000 permutations (blue horizontal line). N = 114 F_4:5_ families.

**Fig 3:**
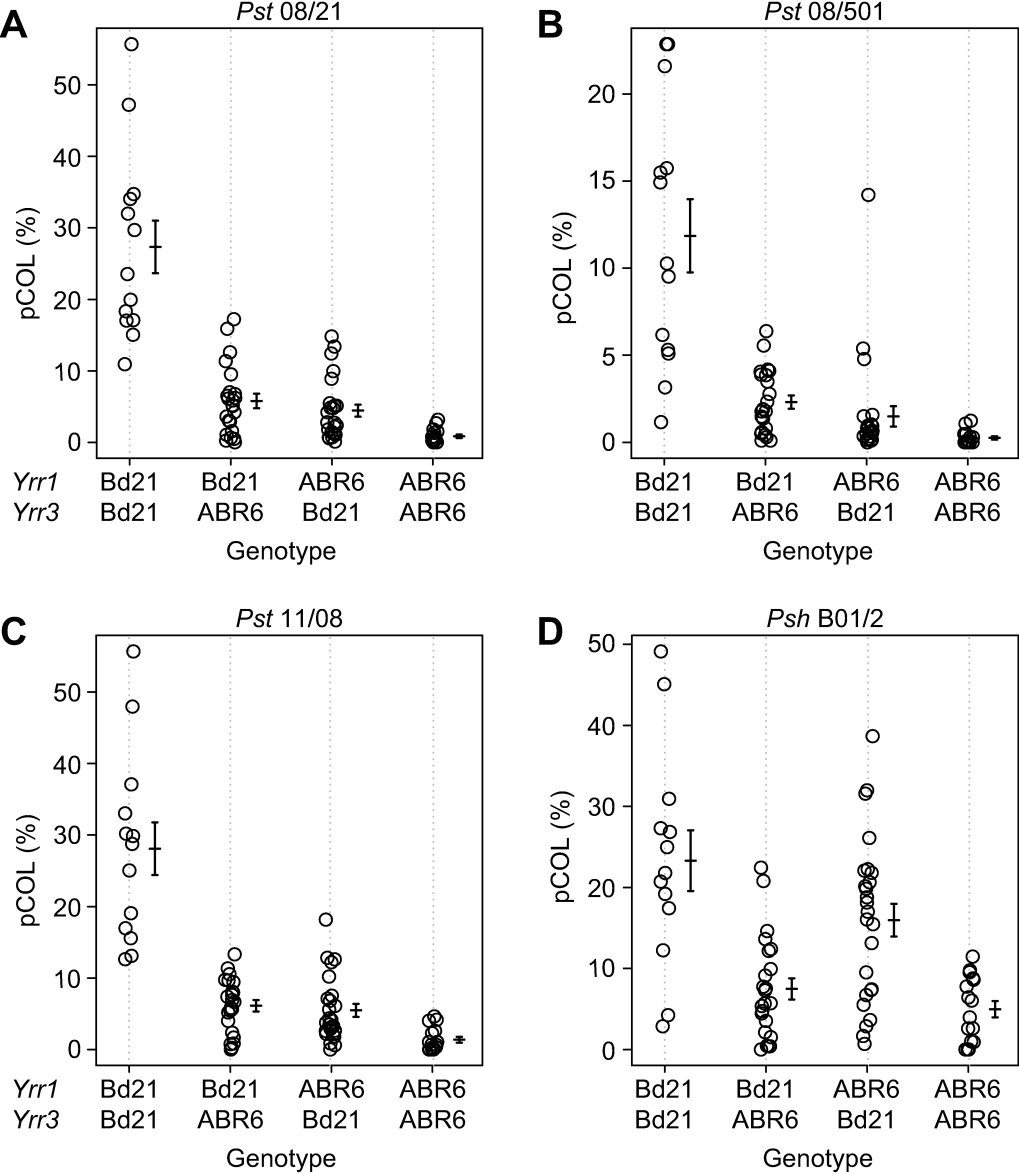
Restriction of *P*. *striiformis* f. sp. *tritici* and *P*. *striiformis* f. sp. *hordei* colonization in the ABR6 x Bd21 F_4:5_ population by *Yrr1* and *Yrr3*. Phenotype by genotype for the major effect loci *Yrr1* and *Yrr3* for *Pst* isolates 08/21 (A), 08/501 (B), and 11/08 (C), and *Psh* isolate B01/2 (D). pCOL phenotypes for lines homozygous at *Yrr1* (marker Bd4_29700796) and *Yrr3* (marker Bd2_51527431) show that ABR6 alleles at both loci provide resistance to *Pst* isolates, whereas only *Yrr3* contributes to resistance against *Psh* isolate B01/2. Error bars represent one standard error. Number of families for the four homozygous groups from left to right: 13, 23, 25, and 16.

Two major effect QTLs were found to control leaf browning and pCOL for *Pst* isolate 08/21 in all three populations. In the ABR6 x Bd21 F_4:5_ population, a QTL at 328.0 cM on chromosome Bd2 controlled 17.8% of the phenotypic variation for leaf browning and 24.0% of the phenotypic variation for pCOL (Fig 2 and Table 1). A second QTL with peak markers located around 140 cM on chromosome Bd4 controlled 10.9% of the variation for leaf browning and 18.3% of the variation for pCOL. These QTLs on chromosomes Bd2 and Bd4 were designated *Yrr3* and *Yrr1*, respectively (Fig 3). Only one additional minor effect QTL was detected for *Pst* isolate 08/21, which explained 4.5% of the phenotypic variation for pCOL in the first replicate (S7A Fig and S1 Table), but was not detected in the second replicate or the averaged dataset. All statistically significant QTLs were contributed by the resistant parent ABR6. Two-dimensional QTL analysis only uncovered *Yrr1* and *Yrr3*, which have a non-additive interaction for leaf browning (*p* = 8.9e-8) and pCOL (p = 6.3e-8) (Fig 3 and S1 File).

**Table 1:** Significant QTLs from composite interval mapping of averaged leaf browning and percent colonization phenotypes for *P*. *striiformis* isolates in the ABR6 x Bd21 F_4:5_ population.

Isolate^1^	Trait^2^	Locus	Chr^3^	cM	EWT^4^	LOD	AEE^5^	PVE^6^
*Pst* 08/21	Browning	*Yrr3*	Bd2	328.0	2.71	6.21	-0.159	17.8
*Pst* 08/21	Browning	*Yrr1*	Bd4	142.8	2.71	5.60	-0.130	10.9
*Pst* 08/21	pCOL	*Yrr3*	Bd2	328.0	2.78	10.95	-0.054	24.0
*Pst* 08/21	pCOL	*Yrr1*	Bd4	139.7	2.78	10.27	-0.049	18.3
*Pst* 08/501	Browning	*Yrr3*	Bd2	328.0	2.87	10.19	-0.317	24.6
*Pst* 08/501	Browning	*Yrr1*	Bd4	144.8	2.87	8.57	-0.235	21.7
*Pst* 08/501	pCOL	*Yrr3*	Bd2	328.0	3.00	8.34	-0.025	19.4
*Pst* 08/501	pCOL	*Yrr2*	Bd4	92.1	3.00	5.31	-0.020	11.1
*Pst* 08/501	pCOL	*Yrr1*	Bd4	139.7	3.00	8.05	-0.024	17.2
*Pst* 11/08	Browning	*Yrr3*	Bd2	328.0	2.61	6.02	-0.103	15.6
*Pst* 11/08	Browning	*Yrr1*	Bd4	142.8	2.61	6.70	-0.105	15.6
*Pst* 11/08	pCOL	*Yrr3*	Bd2	328.0	2.86	11.94	-0.051	23.0
*Pst* 11/08	pCOL	*Yrr2*	Bd4	89.2	2.86	3.25	-0.026	4.5
*Pst* 11/08	pCOL	*Yrr1*	Bd4	139.7	2.86	9.66	-0.043	14.9
*Pst* 11/08	pCOL	–	Bd5	74.3	2.86	3.27	-0.025	5.3
*Psh* B01/2	Browning	–	Bd2	169.8	3.11	3.43	0.168	11.8
*Psh* B01/2	Browning	*Yrr3*	Bd2	326.2	3.11	10.32	-0.250	28.3
*Psh* B01/2	pCOL	*Yrr3*	Bd2	328.9	3.14	10.50	-0.064	27.3
*Psh* B01/2	pCOL	–	Bd3	323.0	3.14	3.98	-0.031	8.0

^1^*Puccinia striiformis* isolate (*Pst* = f. sp. *tritici*, *Psh* = f. sp. *hordei*)

^2^Browning = leaf browning; pCOL = percent colonization

^3^Chromosome

^4^Experiment-wide permutation threshold

^5^Additive effect estimate

^6^Percent of variation explained

**Fig 4:**
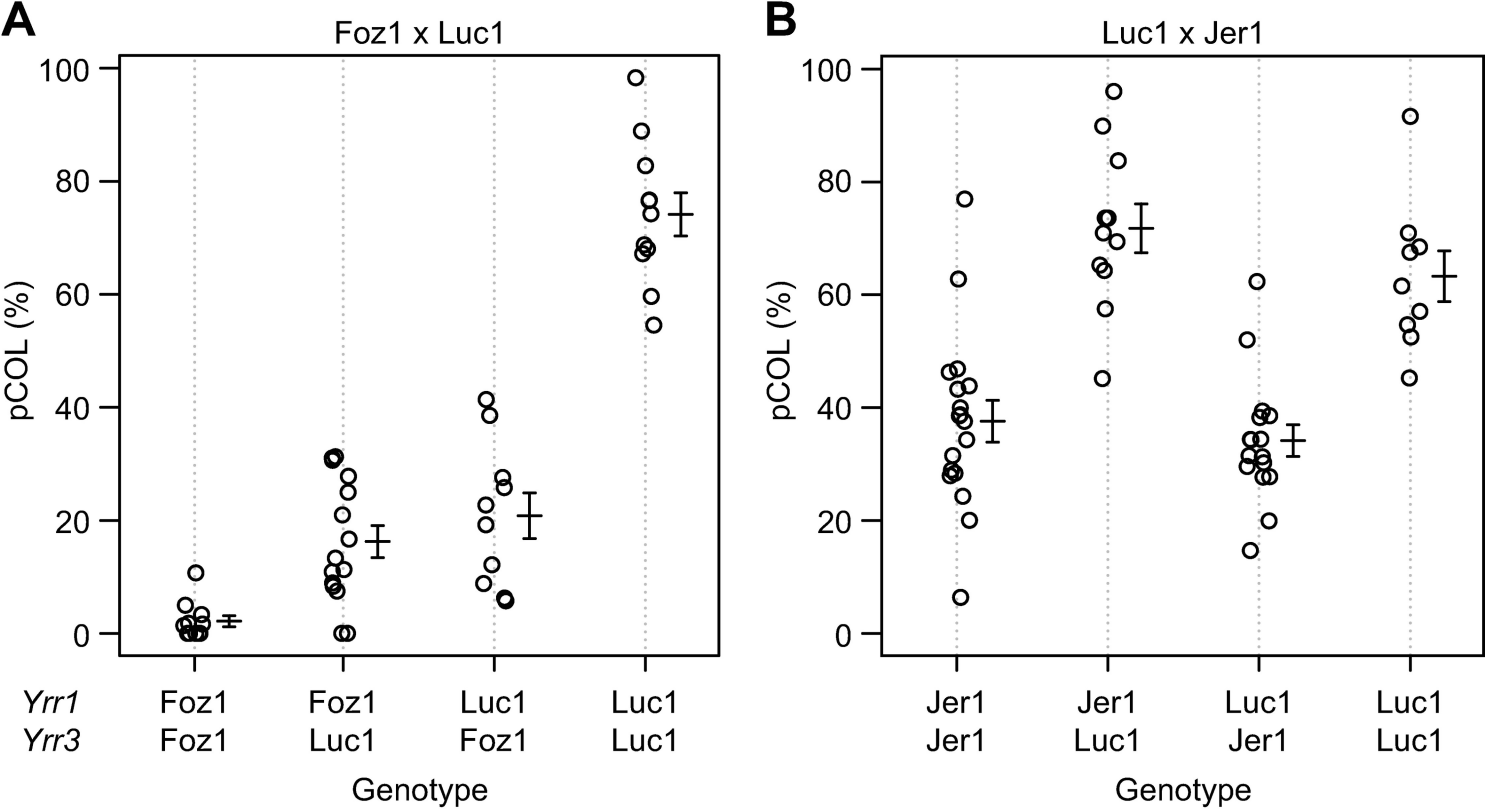
Restriction of *P*. *striiformis* f. sp. *tritici* colonization in Foz1 x Luc1 and Luc1 x Jer1 F_2_ populations by *Yrr1* and *Yrr3*. Phenotype by genotype for the major effect loci *Yrr1* and *Yrr3* in the Foz1 x Luc1 (A) and Luc1 x Jer1 (B) F_2_ populations. pCOL phenotypes for lines homozygous at *Yrr1* (marker Bd4_29128782_60_R) and *Yrr3* (markers Bd2_51602833_60_F (A) and Bd2_51552779_60_F (B)) show that Foz1 alleles at both loci provide resistance in the Foz1 x Luc1 F_2_ population, whereas only *Yrr3* contributes to resistance in the Luc1 x Jer1 F_2_ population. Error bars represent one standard error. Number of individuals for the four homozygous groups from left to right: (A) 11, 15, 10, and 11; (B) 18, 11, 16, and 9.

The same two major effect QTLs, *Yrr1* and *Yrr3*, also segregated in the Foz1 x Luc1 F_2_ population (Fig 4). However, unlike the ABR6 x Bd21 F_4:5_ population, *Yrr1* was the sole major effect QTL that controlled leaf browning, whereas both *Yrr1* and *Yrr3* controlled pCOL (Table 2 and S8A Fig). *Yrr1* accounted for 37.4% and 48.9% of the variation observed in the population (peak markers near 100 cM) and *Yrr3* controlled 28.2% of the variation observed for pCOL. A minor effect QTL on chromosome Bd4 contributed to the pCOL phenotype, accounting for 8.2% of the variation observed (Table 2). All three QTLs were contributed by the resistant parent Foz1. Similar to the ABR6 x Bd21 F_4:5_ population, *Yrr1* and *Yrr3* had a non-additive interaction in the Foz1 x Luc1 F_2_ population (Fig 4 and S1 File).

**Table 2:** Significant QTLs from composite interval mapping of leaf browning and percent colonization phenotypes for *P*. *striiformis* f. sp. *tritici* isolate 08/21 in the Foz1 x Luc1 and Luc1 x Jer1 F_2_ populations.

Population	Trait^1^	dpi^2^	Locus	Chr^3^	cM	EWT^4^	LOD	AEE^5^	DEE^6^	D/A^7^	PVE^8^
Foz1xLuc1	Browning	14	*Yrr1*	Bd4	100.4	16.97	30.15	-0.638	-0.492	0.77	42.9
	Browning	23	*Yrr1*	Bd4	100.4	15.70	36.84	-0.979	-0.707	0.72	48.9
	pCOL	23	*Yrr3*	Bd2	274.8	3.76	20.66	-0.155	-0.021	0.13	28.2
	pCOL	23	*Yrr2*	Bd4	69.3	3.76	4.08	-0.003	-0.120	46.27	8.2
	pCOL	23	*Yrr1*	Bd4	98.4	3.76	27.40	-0.182	-0.091	0.50	37.4
Luc1xJer1	Browning	14	*Yrr3*	Bd2	263.3	3.74	28.40	0.946	-0.612	-0.65	46.5
	Browning	23	–	Bd1	213.0	3.64	4.00	-0.451	0.191	-0.42	7.3
	Browning	23	*Yrr3*	Bd2	258.6	3.64	16.05	1.085	-0.355	-0.33	27.2
	Browning	23	–	Bd3	236.7	3.64	3.91	0.419	0.058	0.14	6.0
	pCOL	23	*Yrr3*	Bd2	263.3	3.51	26.44	0.163	-0.074	-0.45	40.4
	pCOL	23	–	Bd3	50.5	3.51	7.53	0.077	-0.002	-0.02	10.3
	pCOL	23	*Yrr2*	Bd4	87.4	3.51	9.40	-0.092	-0.018	0.19	14.5
	Browning^9^	14	*Yrr3*	Bd2	260.6	3.89	15.10	0.517	-0.126	-0.24	41.3
	Browning^9^	14	–	Bd5	96.2	3.89	4.54	0.063	-0.332	-5.28	11.1

^1^Browning = leaf browning; pCOL = percent colonization

^2^Days post inoculation

^3^Chromosome

^4^Experiment-wide permutation threshold

^5^Additive effect estimate

^6^Dominance effect estimate

^7^Estimate of dominance to additivity ratio

^8^Percent of variation explained

^9^F_2:3_ derived families phenotyped

In contrast to the ABR6 x Bd21 F_4:5_ and Foz1 x Luc1 F_2_ populations, only one major effect QTL conferred resistance in the Luc1 x Jer1 F_2_ population (Figs 4B and S8). *Yrr3* explained between 27.2% and 46.5% of the variation observed for the four phenotypes (Table 2). The physical location of the QTL corresponds to the same locus observed in the ABR6 x Bd21 F_4:5_ population. Several minor effect QTLs were only detected in individual replicates. With the exception of the minor effect QTLs on the long arm of chromosome Bd1 and the short arm of chromosome Bd4, all QTLs were contributed by the resistant parent Jer1. Therefore, only two major effect QTLs were identified in the three mapping populations in response to *Pst* isolate 08/21.

### *Yrr1* and *Yrr3* confer resistance to diverse *Pst* isolates in the ABR6 x Bd21 F_4:5_ mapping population

To investigate the conservation of *Yrr1* and *Yrr3* in colonization resistance to diverse *Pst* isolates, the ABR6 x Bd21 F_4:5_ population was inoculated with the UK *Pst* isolates 08/501 and 11/08. These isolates are genetically distinct to isolate 08/21 and have differential infection outcomes on wheat accessions with various *Yr* resistance genes [38].

Similar to the phenotypic distributions observed for *Pst* isolate 08/21, the infection phenotypes were heavily skewed towards resistance and a strong correlation between leaf browning and pCOL was observed (S1D–S1I Fig). Linkage analyses with the leaf browning phenotype identified *Yrr1* and *Yrr3* as the two major effect QTLs for both isolates (Figs 2B, 2C, 3B and 3C and Table 1). *Yrr1* accounted for 21.7% and 15.6% of the phenotypic variation observed upon infection with *Pst* isolates 08/501 and 11/08, whereas *Yrr3* was responsible for 24.6% and 15.6% of the phenotypic variation for these two isolates. No additional QTLs were identified in individual replicate experiments (S7C–S7F Fig and S1 Table). These two QTLs also had major effects on pCOL, with *Yrr1* contributing 17.2% and 14.9% and *Yrr3* contributing 19.4% and 23.0% of the phenotypic variation for *Pst* isolates 08/501 and 11/08, respectively.

The greater resolution obtained with the pCOL phenotype enabled the identification of two additional minor effect QTLs that exhibited isolate specificity. A QTL on the short arm of chromosome Bd4 accounted for 4.5% of the variation for *Pst* isolate 11/08 and 11.1% of the variation for *Pst* isolate 08/501 (Fig 2B and 2C and Table 1). As this QTL was statistically significant for more than one *Pst* isolate tested, it was designated *Yrr2*. A QTL on chromosome Bd5 was only statistically significant for *Pst* isolate 11/08 and explained 5.3% of the phenotypic variation (Fig 2C and Table 1). Two-dimensional QTL analysis using the pCOL phenotype for *Pst* isolates 08/501 and 11/08 found significant pair-wise non-additive interactions between *Yrr1*, *Yrr2*, and *Yrr3* (S1 File). Collectively, these results indicate that *Yrr1* and *Yrr3* contribute to colonization resistance to all *Pst* isolates tested, whereas *Yrr2* exhibited isolate specificity in its detection.

### Broad-spectrum and isolate-specific resistance to *P*. *striiformis* in *B*. *distachyon*

Using three diverse *Pst* isolates in the ABR6 x Bd21 F_4:5_ population, we found no evidence of isolate specificity for *Yrr1* or *Yrr3*. To determine if these major effect loci are specific for *Pst* or also provide broader resistance to other *P*. *striiformis formae speciales*, the mapping population was challenged with the barley-adapted *Psh* isolate B01/2. Similar to *Pst*, phenotypes obtained for *Psh* were not normally distributed and skewed towards resistance for both leaf browning and pCOL (S1J and S1K Fig). Transgressive segregation was observed with some F_4:5_ families displaying increased leaf browning and pCOL compared to Bd21. In contrast to the three *Pst* isolates tested, ABR6 displayed some macroscopic *Psh* infection symptoms with an average leaf browning score of 0.3 and very limited hyphal colonization (pCOL of 2%). Leaf browning and pCOL phenotypes were correlated with a correlation coefficient of 0.63 (S1L Fig).

Unlike resistance to *Pst* in this population, resistance to *Psh* was predominantly due to a single locus that colocalized with *Yrr3* (Fig 2D). This locus explained 28.3% and 27.3% of the phenotypic variation for leaf browning and pCOL, respectively (Fig 3D and Table 1). No statistically significant QTLs were observed on chromosome Bd4 using averaged data (Fig 2D) or individual replicates (S7G and S7H Fig). Chromosome Bd4 harbors the major effect locus *Yrr1* and the minor effect locus *Yrr2*, which both confer resistance to *Pst* isolates. While *Yrr3* possesses greater recognition capability towards another *P*. *striiformis forma specialis*, *Yrr1* and *Yrr2* appear to specifically recognize *Pst* isolates only.

### The *Yrr3* locus is tightly linked with a cluster of NB-LRR encoding genes

Similarity with host systems in the form of major effect loci and isolate specificity prompted us to check the gene content of these QTLs. Several classes of plant immune receptors confer resistance to adapted pathogens, including NB-LRR, kinase-kinase, and LRR-kinase encoding genes [39, 40]. To date, the majority of cloned resistance genes from host pathosystems encode NB-LRR proteins [41, 42]. To determine whether NB-LRR are associated with *Yrr1* and *Yrr3* loci, we evaluated the one-LOD and two-LOD support intervals defined by interval mapping with the pCOL phenotypes (S2 File) and examined the gene content of these regions (S3 and S4 Files). Annotated gene models in the *Yrr1* and *Yrr3* intervals were characterized by comparing the Bd21 reference genome with resequencing data from ABR6, Luc1, and Jer1 [36]. Gene expression was assessed by aligning RNAseq reads to the updated gene models from each accession (S3 and S4 Files).

Of the 677 genes that were predicted in the *Yrr1* interval, most have a resequencing read alignment coverage above 90% (Table 3). Most gene models present in ABR6 (58%), Luc1 (76%), and Jer1 (71%) encoded nonsense or non-synonymous substitutions when compared with the Bd21 reference sequence. No transcripts were detected for 124 (ABR6) to 152 (Jer1) of these gene models, while 74 gene models were not expressed in any of the four accessions. A similar situation was observed for the 789 gene models predicted in the *Yrr3* interval. Again, high sequence coverage was obtained for most gene models (Table 3) and many gene models in ABR6 (44%), Luc1 (44%), and Jer1 (42%) contained nonsense or non-synonymous substitutions when compared with Bd21. Transcripts were identified for the vast majority of the gene models in the *Yrr3* interval.

**Table 3:** Analysis of annotated gene models within the maximal two-LOD support intervals of *Yrr1* and *Yrr3*. Numbers represent the number of gene models within each category.

Locus	Accession	Alignment coverage < 50%	Alignment coverage > 90%	InDel^1^	SNP^2^	Modified stop codon^2^	Amino acid change^3^	Not expressed^4^
*Yrr1*:	Bd21^5^	–	–	–	–	–	–	133
677 gene	ABR6	14	637	35	395	26	334	124
models	Luc1	39	620	43	517	40	434	150
	Jer1	25	633	19	509	36	425	152
*Yrr3*:	Bd21	–	–	–	–	–	–	58
789 gene	ABR6	0	788	8	412	4	334	62
models	Luc1	0	789	12	404	2	330	76
	Jer1	0	784	8	406	3	323	76

^1^Insertion or deletion in coding sequence relative to Bd21 reference

^2^Of gene models without InDels

^3^Of gene models without InDels or modified stop codons

^4^No RNAseq reads aligned to gene model

^5^Only expression assessed for Bd21

**Fig 5:**
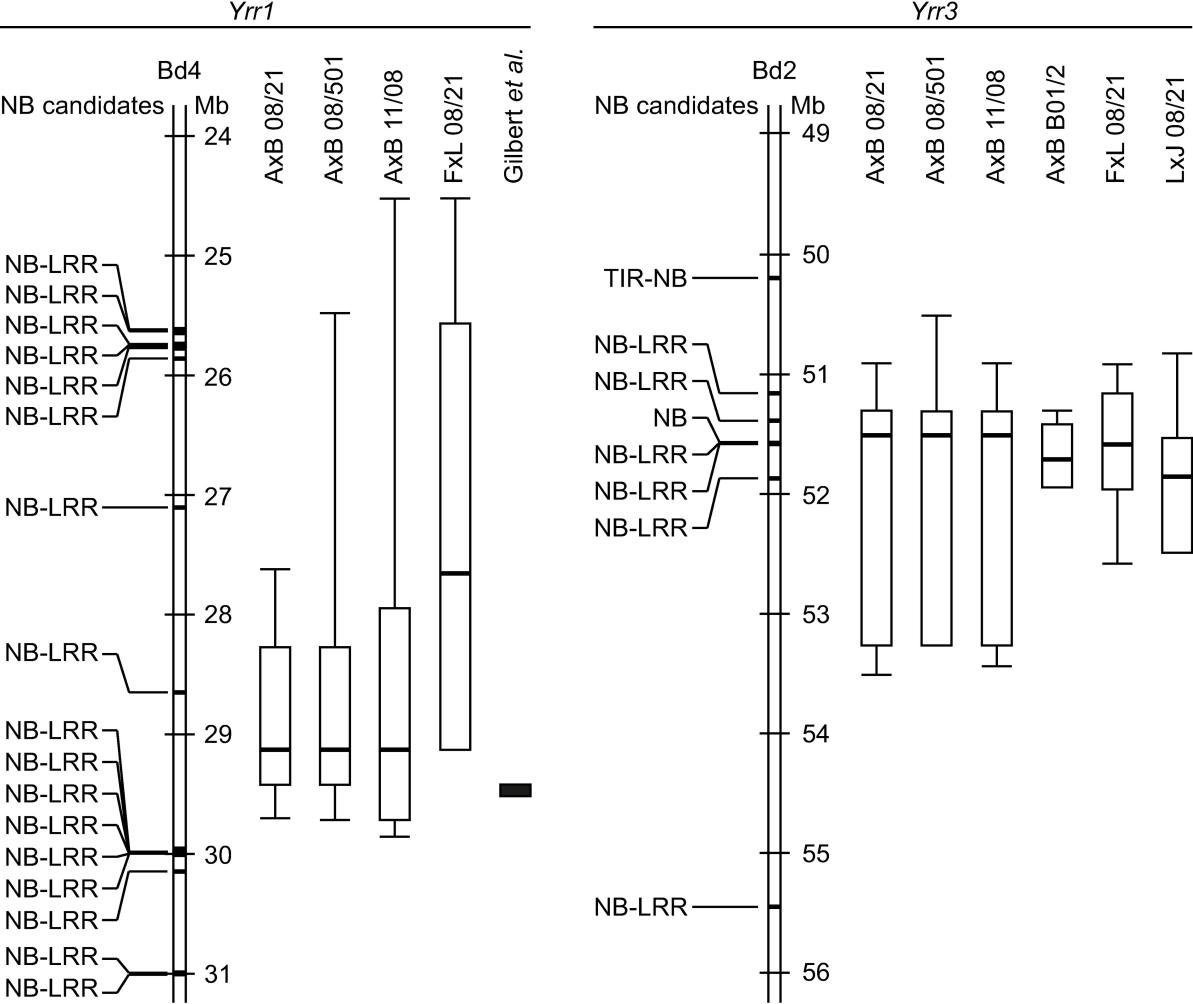
Canonical resistance genes associated with the *Yrr1* and *Yrr3* loci. Annotated nucleotide binding (NB) domain or leucine-rich repeat (LRR) encoding genes from the Bd21 reference sequence are indicated. One-LOD and two-LOD support intervals for pCOL phenotypes were determined with interval mapping. Within boxplots, thick bars denote the peak marker; the box defines the one-LOD support interval, and whiskers delineate the two-LOD support interval. Missing whiskers indicate a shared one-LOD and two-LOD support interval boundary. The solid black box corresponds to the fine-mapped *Yrr1* interval (see Gilbert *et al*.). Gene identifiers for the canonical resistance genes are listed in S5 File. Structural variation and gene expression information can be found in S6 File. AxB = ABR6 x Bd21 F_4:5_ population; FxL = Foz1 x Luc1 F_2_ population; LxJ = Luc1 x Jer1 F_2_ population; 08/21, 08/501, and 11/08 = *Pst* isolates; B01/2 = *Psh* isolate.

Both the *Yrr1* and *Yrr3* loci contained clusters of NB-LRR encoding genes (Fig 5 and S6 File). However, no strong linkage was observed between the NB-LRR encoding genes at the *Yrr1* locus (S6 File) and subsequent fine-mapping confirmed the lack of NB-LRR candidates for this locus (see Gilbert *et al*.). In contrast, the combined maximal two-LOD support interval for the pCOL phenotypes at *Yrr3* contains five NB-LRR encoding genes and one NB domain encoding gene. These were expressed in all accessions and with the exception of one also possess structural variation in the other three *B*. *distachyon* accessions (S6 File). Crucially, the *Yrr3* peak markers center around a cluster of two NB-LRR genes (Bradi2g52437 and Bradi2g52450) and a gene encoding an NB domain only (Bradi2g52430) (Fig 5 and S6 File). These data suggest the involvement of NB-LRR encoding genes in *Yrr3* resistance, whereas their involvement in *Yrr1* mediated resistance remains unclear.

### The *P*. *striiformis* isolates and *formae speciales* are phylogenetically distinct

The significance of broad-spectrum or isolate-specific effectiveness of resistance is dependent on the genetic diversity of the *P*. *striiformis* isolates used. The three *Pst* isolates used in our study are known to have different avirulence specificities on wheat accessions with various *Yr* genes [38]. In addition, the two isolates identified in 2008 (08/21 and 08/501) are more closely related, whereas the 2011 isolate (11/08) represents a later incursion of *Pst* into the UK [38]. To understand the genetic relationships of *P*. *striiformis* isolates and *formae speciales* used in this study, we set out to develop a phylogenetic tree based on transcriptomic and genomic resources.

We sequenced the transcriptome of barley leaves infected with *Psh* isolate B01/2 and used publicly available transcriptome or whole genome sequencing datasets for the three UK *Pst* isolates [38], the Australian *Pst* isolate 104E137A- [43], and the reference genome of the US *Pst* isolate 78 [44]. Of the annotated genes in the *Pst* 78 reference genome, 546 genes spanning 562,662 bp had sufficient coverage in all datasets. Pairwise sequence comparisons of these genes showed that the two 2008 UK *Pst* isolates, 08/21 and 08/501, are almost identical in sequence for these genes and highly similar to the Australian *Pst* isolate 104E137A- (Table 4). The US *Pst* isolate 78 and the UK *Pst* isolate 11/08 are more diverged from these three isolates. However, consistent with the *formae speciales* divide, *Psh* isolate B01/2 is the most divergent isolate in our analysis. These pairwise sequence comparisons were supported by calculating the substitution rates and building a phylogenetic tree of the six *P*. *striiformis* isolates using maximum likelihood (Table 4 and S9 Fig). These analyses demonstrate that *Yrr3* is a broad-spectrum QTL that recognizes highly divergent *P*. *striiformis* isolates.

**Table 4:** Pairwise comparison of polymorphic sites (top) and patristic distances of nucleotide substitutions per million sites shown in S9 Fig (bottom) between the different *P*. *striiformis* isolates.

	*Pst* 78^1^ (DNA)	*Pst* 08/21 (DNA)	*Pst* 08/501 (DNA)	*Pst* 104E137A- (RNA)	*Pst* 11/08 (DNA)	*Pst* 11/08 (RNA)	*Psh* B01/2 (RNA)
*Pst* 78 (DNA)	–	164	163	180	331	332	472
*Pst* 08/21 (DNA)	295	–	1	18	263	270	388
*Pst* 08/501 (DNA)	296	3	–	19	264	271	389
*Pst* 104E137A- (RNA)	320	33	34	–	273	262	392
*Pst* 11/08 (DNA)	698	485	486	510	–	21	519
*Pst* 11/08 (RNA)	702	489	490	514	38	–	518
*Psh* B01/2 (RNA)	933	720	721	745	925	929	–

^1^*Puccinia striiformis* isolate (*Pst* = f. sp. *tritici*, *Psh* = f. sp. *hordei*)

## Discussion

In dissecting the genetic architecture of resistance in *B*. *distachyon* to several diverse *P*. *striiformis* isolates at the colonization stage, we identified a genetic architecture that is similar to a host system. Two major effect loci were identified, *Yrr1* and *Yrr3*, which confer colonization resistance to diverse *P*. *striiformis* isolates. Previous work in rice found limited natural variation in resistance to *P*. *striiformis* [31]. Therefore, *B*. *distachyon* is the most phylogenetically distant grass to wheat and barley for which the genetic basis of *P*. *striiformis* resistance has been dissected. The genetic architecture of colonization resistance in this system displayed hallmarks of the genetic architecture of resistance in adapted systems: major effect loci and isolate specificity.

### The genetic architecture of resistance to *P*. *striiformis* in *B*. *distachyon* resembles resistance to adapted pathogens

Nonhost resistance is defined as all accessions from a plant species being resistant to all isolates of a particular pathogen [45, 46]. For example, rice is considered a nonhost of rusts, as no naturally susceptible rice accessions have been identified [31–33]. A rice mutant that allows some *Pst* colonization has recently been described [47], but in the absence of natural or induced variation interspecific crosses may be the last genetic approach at dissecting nonhost resistance. Such experiments are often prevented by interspecies sexual incompatibility and limited by our ability to cross plants [48]. To dissect resistance in phylogenetically more distant species, it is therefore necessary to study resistance within species that fall onto the continuum from host to nonhost, i.e. species in which some accessions allow a degree of infection or colonization, but other accessions are resistant [30, 48, 49].

While most *B*. *distachyon* accessions possess barriers against *P*. *striiformis* colonization, a subset of accessions allows leaf colonization, before additional barriers prevent further disease progression [28]. Several researchers have proposed that the genetic architecture and molecular basis of resistance to non-adapted pathogens are fundamentally different from the gene-for-gene interactions observed in host systems [48, 50, 51]. In our study, the two major effect QTLs *Yrr1* and *Yrr3* control colonization in response to the *Pst* isolates, whereas only *Yrr3* was detected in response to *Psh*. Our findings therefore highlight a genetic architecture that relies on major effect loci. In the case of *Yrr1* the major effect locus also displays isolate specificity. Both of these characteristics are commonly attributed to resistance against adapted pathogens.

Barbieri *et al*. [34] studied the interaction between *B*. *distachyon* and the adapted rust *P*. *brachypodii*. In a mapping population derived from the accessions Bd3-1 and Bd1-1 the authors identified the loci preventing pustule formation. Analyses of the F_2_ population and F_2:3_ families found three QTLs, two of which govern resistance at the seedling stage and one which governs resistance at the seedling stage and an advanced growth stage. Ayliffe *et al*. [27] studied the inheritance of resistance to the Australian *Pst* isolate 104E137A- in an F_4_ population (BdTR13k x Bd21) and an F_2_ population (BdTR10h x Tek-4). The authors assessed the extent of pathogen growth based on macroscopic lesions and occasionally also observed pustule formation in the segregating progeny. The segregation ratios of infection symptoms suggested a simple genetic architecture of two genes and one gene restricting pathogen growth in these populations. Subsequent linkage analyses have identified these loci as *Yrr1* and *Yrr2* (see Gilbert *et al*.). Taken together, our results challenge existing assumptions about the genetic basis of resistance [48, 50–55] and support a genetic model of an overlap between resistance to adapted and non-adapted pathogens [3, 56, 57].

### No regular life cycle completion of *P*. *striiformis* in *B*. *distachyon*

Extensive diversity exists within barley for the entire range of resistance and susceptibility symptoms following *Pst* infection [28]. These include complete immunity, varying degrees of chlorosis associated with hyphal colonization, and pustule formation in the absence of chlorosis (as observed in the adapted interaction between *Pst* and wheat). In contrast, only complete immunity and hyphal colonization were observed in *B*. *distachyon*. In a diversity panel of 210 *Brachypodium* spp. accessions, pustule formation was largely limited to the close allotetraploid relative *B*. *hybridum* [28]. Our study of three mapping populations incorporated phenotypically and genetically diverse *B*. *distachyon* accessions [36] and diverse *P*. *striiformis* isolates. The parental accessions never exhibited pustule formation in our experiments and we only very rarely observed pustule formation in the progeny. Consequently, no phenotypic assay was developed to assess life cycle completion.

The multiple barriers to successful pathogen life cycle completion are highlighted by the lack of regular life cycle completion in the transgressively segregating *B*. *distachyon* mapping populations. The absence of regular pustule formation shows that even in plants with extensive colonization an additional layer of incompatibility prevents life cycle completion. As the interaction between plant and pathogen is complex, this could be due to the inability of the pathogen to modify the plant in the same manner as the adapted host. During colonization of an adapted host, pathogens secrete effectors to alter the host environment and facilitate infection [11, 12]. In this scenario, lack of pustule formation could be due to the inability of *P*. *striiformis* to create conducive conditions for the transition from growth to reproduction. Alternatively, the pathogen may lack appropriate host plant signals or cues to initiate life cycle progression. Life cycle progression could also be prevented by an active, induced defense response, which would hint at an absence of variation in the gene or genes limiting pustule formation among the *B*. *distachyon* accessions studied. In barley, natural variation exists for resistance to *P*. *striiformis* that limits the pathogen at pustule formation, but not hyphal colonization [28]. Therefore, it is possible that a conserved gene in *B*. *distachyon* may limit the lifecycle completion of *P*. *striiformis*.

### The molecular basis of resistance on the continuum from host to nonhost systems

The arms race between host plant and adapted pathogens has resulted in the evolution of numerous resistance genes that often only confer resistance to particular pathogen isolates. Historically, this allowed Biffen to demonstrate that resistance to *P*. *striiformis* in wheat follows Mendel’s laws [58]. Many wheat and barley genes that confer resistance to *Pst* and *Psh* isolates have been mapped (see Chen (20) for a review of *Pst* resistance loci in wheat). These single resistance genes in host systems have often been identified as NB-LRR encoding genes and act in an isolate-specific manner towards the pathogen [41, 59]. While the role of NB-LRRs in resistance to adapted pathogens is accepted, it remains unclear (1) how resistance to non-adapted pathogens is maintained in the absence of selection in plants phylogenetically distant to the adapted host and (2) the capacity of plant immune receptors to contribute to resistance against non-adapted pathogens.

Remarkably, we observed characteristics typical for resistance to adapted pathogens in resistance to non-adapted pathogens. Namely, these included the identification of major effect genes, isolate specificity for both major and minor effect QTLs, and NB-LRR gene clusters associated with the identified QTLs. *Yrr1* is a major effect QTL controlling leaf browning and hyphal colonization in response to all three *Pst* isolates tested. However, in the ABR6 x Bd21 F_4:5_ population this QTL does not control resistance in response to *Psh* isolate B01/2. Additionally, all of the minor effect QTLs detected in the ABR6 x Bd21 F_4:5_ population in response to the three *Pst* isolates displayed isolate specificity, although this may be associated with limits of statistical detection. Isolate specificity is a common feature in host-pathogen interactions, due to the gene-for-gene interaction in host systems [16]. ETI exerts considerable selection pressure on pathogen populations, which leads to effector loss or modification to avoid detection by the host plants [13]. The emergence of new isolates with an altered effector repertoire consequently leaves the plant with isolate-specific resistance genes [13]. In line with this, candidate genes encoding NB-LRRs and a phosphatase have been identified for two of the *B*. *distachyon* loci providing resistance to the adapted pathogen *P*. *brachypodii* [60]. As resistance towards non-adapted pathogens is commonly thought to be governed by many, minor effect QTLs reminiscent of basal host resistance [48] we did not expect isolate-specific major effect genes to control the interaction between *B*. *distachyon* and *Pst* and *Psh* isolates. Of particular interest is the observation that fine-mapping of *Yrr1* did not uncover a known class of plant immune receptor, despite exhibiting isolate specificity (see Gilbert *et al*.). In contrast, tight linkage observed between peak markers at *Yrr3* and an NB-LRR cluster opens the possibility that these canonical host immune receptors may contribute to *Pst* and *Psh* resistance in *B*. *distachyon* [61].

### A shared genetic architecture for resistance to adapted and non-adapted pathogens

While it has been proposed that resistance to adapted and non-adapted pathogens is inherently different, the genetic architecture of colonization resistance in this intermediate nonhost system is reminiscent of a host system. Moreover, the isolate specificity observed for major and minor effect QTLs and the associated NB-LRR encoding candidate genes suggest that the genetic architectures of resistance to adapted and non-adapted pathogens are structurally coupled and share conserved components. Emphasis has been placed on the intrinsic differences between resistance to adapted and non-adapted pathogens, whereas resistance to non-adapted pathogens may reflect a complete form of resistance, which can draw on a wide range of barriers to limit pathogen ingress and life cycle progression. In the highly-specialized interaction between a host plant and an adapted pathogen, most of these have been overcome and plant and pathogen are left in an evolutionary arms race where the predominant mechanisms of resistance exhibit major effect and isolate specificity.

## Materials and methods

### Plant and fungal material

The ABR6 x Bd21 F_4:5_ population has been described previously [35]. Seeds for the *B*. *distachyon* accessions Luc1, Jer1, and Foz1 were kindly provided by Luis A.J. Mur (Aberystwyth University), and F_1_ plants were confirmed with CAPS markers (S2 Table). To increase F_2_ seed yield, F_1_ plants were grown in a prolonged vegetative state to increase biomass before vernalization and flowering [62]. F_2_ lines were grown from a single cross for both Luc1 x Jer1 and Foz1 x Luc1. Tissue for DNA extraction and genetic map construction was collected after phenotyping. *P*. *striiformis* isolates were collected in the United Kingdom in 2001 (*Psh* B01/2), 2008 (*Pst* 08/21 and 08/501), and 2011 (*Pst* 11/08). Isolates were maintained at the National Institute of Agricultural Botany on susceptible barley and wheat cultivars, respectively, and urediniospores were stored at 6°C after collection.

### Development of the Luc1 x Jer1 and Foz1 x Luc1 genetic maps

Resequencing data was obtained from the Joint Genome Institute Genome Portal (http://genome.jgi.doe.gov/) for the projects 1000598 (Luc1), 404166 (Jer1), and 404167 (Foz1) [36]. These sequence data were produced by the US Department of Energy Joint Genome Institute (http://www.jgi.doe.gov/) in collaboration with the user community. *De novo* assemblies were created from the raw reads using default settings and parameters of the CLC Assembly Cell (version 4.2.0). To ensure an equal genetic distribution across the whole genome, marker positions were selected based on the ABR6 x Bd21 genetic map [35]. A BLAST search was performed with Bd21 sequence based on desired position against the Luc1, Jer1, and Foz1 *de novo* assemblies. The contig sequences for the respective top hits were aligned in Geneious (version 7.1.8). SNPs without additional sequence variation in a 160 bp window were selected for KASP marker development (S7 File). To confirm the relative position of the Luc1 x Jer1 and Foz1 x Luc1 markers in the Bd21 reference sequence, a BLAST search was performed with the sequences used for KASP marker development. Markers were named according to the relative SNP position in the Bd21 reference sequence (version 3.1). DNA was extracted from leaf tissue of the phenotyped F_2_ lines using a CTAB gDNA extraction protocol modified for plate-based extraction [63]. The final Foz1 x Luc1 genetic map is based on 179 genotyped F_2_ lines and contains 101 non-redundant markers (S8 File). The final Luc1 x Jer1 genetic map is based on 188 genotyped F_2_ lines and contains 107 markers (S9 File). The quality of the genetic maps was confirmed by analyzing recombination fractions in R/qtl (version 1.33–7) and segregation distortion was assessed using chi-square tests with Bonferroni correction for multiple comparisons.

### Plant growth, inoculation, and phenotyping

For the ABR6 x Bd21 population, 114 F_4:5_ families were sown in groups of four in 1 L pots containing peat-based compost. For the Foz1 x Luc1 and Luc1 x Jer1 F_2_ populations, 188 F_2_ individuals were sown individually in 24-hole trays containing peat-based compost. Plants were grown at 18°C day and 11°C night in a 16 h photoperiod in a controlled environment room. Seedlings were inoculated four weeks after sowing at the four to five leaf stage as described previously [28]. In the ABR6 x Bd21 F_4:5_ population, leaf browning and pCOL phenotypes were scored at 14 dpi [28]. Phenotypes were scored for each individual in a family and then averaged (S10 File). The two *Pst* 08/501 replicates consisted of 20 and five plants per F_4:5_ family, respectively. The two *Pst* 08/21 replicates consisted of 10 and five plants per F_4:5_ family, respectively. All replicates of *Pst* 11/08 and *Psh* B01/2 consisted of five plants per F_4:5_ family. In the Foz1 x Luc1 and Luc1 x Jer1 F_2_ populations, F_2_ plants were phenotyped individually at 14 dpi for leaf browning and at 23 dpi for leaf browning and pCOL (S11 File). Additionally, 95 Luc1 x Jer1 F_2:3_ families were phenotyped by growing and inoculating 16 F_3_ plants in a 1 L pot. Leaf browning phenotypes were scored at 14 dpi for each individual in a family and then averaged. All experiments were performed using a random complete design. Phenotypes were assessed for normality using the Shapiro-Wilk test [64] and Pearson rank correlation coefficients (ρ) between leaf browning and pCOL phenotypes were determined using the *cor* command in R (v3.2.2).

### Quantitative trait locus analyses

For the ABR6 x Bd21 F_4:5_ population, composite interval mapping was performed using an additive model (H_0_:H_1_) due to the extensive homozygosity observed at the F_4_ stage (~87.5%). For the Foz1 x Luc1 and Luc1 x Jer1 F_2_ populations, composite interval mapping was performed using the model H_0_:H_3_, which includes both additive and dominance effect estimates. QTL Cartographer (version 1.17j) was used for composite interval mapping with the selection of five background markers, a walking speed of 2 cM, and a window size of 10 cM [65–67]. Statistical significance for QTLs was determined by performing 1,000 permutations with reselection of background markers and controlled at α = 0.05 [68, 69]. For the ABR6 x Bd21 F_4:5_ population, QTL analyses were performed with the phenotyping data from the individual replicates, as well averaged replicates for each isolate. For the Foz1 x Luc1 and Luc1 x Jer1 F_2_ populations, QTL analyses were performed with the individual phenotyping scores from the F_2_ individuals and the averaged phenotyping data from the Luc1 x Jer1 F_2:3_ families. One-LOD and two-LOD support intervals were estimated based on standard interval mapping [70]. Two-dimensional QTL analysis was performed using R/qtl (1.40–8) using scantwo with parameters of step size of 2.0 cM, error probability of 0.001, and 128 number of draws for calc.genoprob and sim.geno, with the Haley-Knott method [71], and significant QTLs identified based on 1,000 permutations with α = 0.05.

### RNAseq of Luc1 and Jer1

Transcriptome sequencing for Luc1 and Jer1 was performed as described for ABR6 and Bd21 previously [35]. Briefly, plants were grown in a controlled environment room with 16 h of light at 22°C, and fourth and fifth leaves were harvested as soon as the fifth leaf was fully expanded (approximately four weeks after sowing). RNA was extracted using TRI-reagent (Sigma-Aldrich; T9424) according to the manufacturer’s specifications. TruSeq libraries were generated from total RNA and mean insert sizes were 253 bp and 248 bp for Luc1 and Jer1, respectively. Library preparation and sequencing was performed at The Genome Analysis Centre (Norwich, UK). Sequencing was carried out using 100 bp paired-end reads on an Illumina HiSeq 2500. Luc1 and Jer1 yielded 134,975,912 and 136,308,576 raw reads, respectively.

### Structural variation and candidate gene analysis at *Yrr1* and *Yrr3*

Resequencing data for ABR6 (project 1079483) was obtained from the Joint Genome Institute Genome Portal (see above for details on Luc1 and Jer1) [36]. An identical quality trimming, read alignment, and SNP/InDel calling strategy was applied to the *B*. *distachyon* loci that was used for the *P*. *striiformis* data set. Assessment of structural variation at the *Yrr1* and *Yrr3* loci was made by converting the Bd21 reference genome to the alternate genotype through the identification of single nucleotide and small insertion/deletion variation (S12 File). QKgenome_conversion.py was used with threshold requirements for read coverage was set at 20 reads and allelic variant frequency of greater than 95%. Tophat (version v2.0.9) was used for splice alignment of RNAseq datasets for Bd21, ABR6, Luc1, and Jer1 to their respective converted reference genomes. FeatureCounts (version v1.5.1) with commands “-M -O -t exon” was used to identify the number of RNAseq reads mapping to individual gene models. To identify canonical NB-LRR encoding resistance genes, the most recent Bd21 reference genome annotation (version 3.1) was searched for genes annotated as encoding NB-ARC domains (Pfam PF00931) and/or belong to the LRR gene family (PANTHER PTHR23155). The identified genes were largely consistent with annotations of previous *B*. *distachyon* reference genome versions [72, 73].

### RNAseq of *Psh* B01/2 and *P*. *striiformis* phylogenetic analysis

The susceptible barley accession Aramir (PI 399482) was inoculated with *Psh* B01/2 as described previously [28]. Plants exhibited a McNeal score of 8 (abundant sporulation with chlorosis) [74]. Infected leaves were harvested 12 dpi and flash frozen in liquid nitrogen. RNA was extracted using TRI-reagent (Sigma-Aldrich; T9424) according to the manufacturer’s specifications. TruSeq libraries were generated from total RNA and mean insert sizes were 280 bp. Library preparation and sequencing was performed at The Genome Analysis Centre (Norwich, UK). Sequencing was carried out using 150 bp paired-end reads on an Illumina HiSeq 2500 and yielded 38,636,376 raw reads.

The *Pst* 78 reference sequence assembly and raw sequencing reads were obtained from the Broad Institute (GenBank BioProject PRJNA41279) [44]. The *Pst* 08/21, *Pst* 08/501, and *Pst* 11/08 genome and transcriptome raw sequencing reads were obtained from the GenBank BioProjects PRJNA256347 and PRJNA257181 [38]. The *Pst* 104E137A- Illumina RNAseq reads from germinated spores and haustoria were obtained from GenBank BioProject PRJNA176472 [43].

Illumina reads were quality controlled using Trimmomatic (version 0.33) with the following parameters: ILLUMINACLIP:TruSeq3-PE.fa:2:30:10 LEADING:5 TRAILING:5 SLIDINGWINDOW:4:15 MINLEN:80. Alignments to the *Pst* 78 reference assembly were performed with bwa mem (version 0.7.5a-r405) with default parameters for gDNA samples and Tophat (version 2.0.9) with default parameters was used for splice alignment mapping of RNAseq samples. Samtools (version 0.1.19-96b5f2294a) was used to convert sam into bam files (samtools view) with the requirement that reads mapped in a proper pair (-f2), to sort the bam file (samtools sort), to remove duplicate reads (samtools rmdup), and to generate an mpileup file (samtools mpileup). Coverage of reads was determined using bedtools (version v2.17.0; bedtools genomecov -d -split). SNPs and InDels were called using VarScan (version 2.3.8) with default parameters.

The QKgenome suite (version 1.1.2) of Python scripts were used to identify SNPs from diverse gDNA and RNA sequenced *Pst* and *Psh* isolates. QKgenome_conversion.py was used with the requirement of a read depth of 20 across the entire gene model for all isolates studied. In addition, only the first gene model for each gene was used to avoid duplication of polymorphic sites by including splice variants. SNPs and InDels were called based on a frequency threshold of 90% (i.e. only homokaryotic polymorphisms were included). All genes with InDels that disrupted the coding sequence were not included in the analysis. A multiple sequence alignment of polymorphic sites was generated using QKgenome_phylogeny.py. The phylogenetic tree was constructed with the GTR CAT nucleotide model, rapid hill-climbing algorithm, and 1,000 bootstrap replicates using RAxML (version 8.2.9).

### Accession numbers

Sequencing data were deposited in NCBI under BioProjects PRJNA376485 (*B*. *distachyon*) and PRJNA376252 (barley/*Psh*). Individual RNAseq reads include accession numbers SRR5279889 (Luc1), SRR5279890 (Jer1), SRR5279891 (Foz1), and SRR5277779 (*Psh* B01/2). *De novo* genome assemblies of *B*. *distachyon* were deposited in figshare (https://figshare.com/projects/The_genetic_architecture_of_colonization_resistance_in_Brachypodium_distachyon_to_non-adapted_stripe_rust_Puccinia_striiformis_isolates/29752). The QKgenome suite of Python scripts described in this manuscript has been deposited on GitHub (https://github.com/matthewmoscou/QKgenome).

## Supporting information

S1 Fig Frequency distribution and correlation of leaf browning and pCOL phenotypes in the ABR6 x Bd21 F_4:5_ population inoculated with several isolates of *P*. *striiformis* f. sp. *tritici*. Distribution of leaf browning (A, D, G, and J) and pCOL (B, E, H, and K) and the correlation between these two phenotypes (C, F, I, and L) in the F_4:5_ families averaged across the two replicates for *Pst* isolates 08/21 (A–C), 08/501 (D–F), and 11/08 (G–I), and for *Psh* isolate B01/2 (J–L). Arrows indicate parental phenotypes. ρ = correlation coefficient.
(PDF)

S2 Fig Frequency distribution and correlation of leaf browning and pCOL phenotypes in the Foz1 x Luc1 and Luc1 x Jer1 F_2_ populations inoculated with *P*. *striiformis* f. sp. *tritici* isolate 08/21. Leaf browning phenotypes were collected at 14 dpi (A and E) and at 23 dpi (B and F), and pCOL phenotypes were collected at 23 dpi (C and G). Correlation between leaf browning and pCOL phenotypes at 23 dpi is shown (D and H). Arrows indicate parental phenotypes. dpi = days post inoculation; ρ = correlation coefficient.
(PDF)

S3 Fig Linkage groups of Foz1 x Luc1 genetic map. Cumulative cM distances and SNP marker names are shown to the left and right of each chromosome, respectively. cM distance at the F_2_ stage was estimated using the Kosambi function. SNP marker names consist of the corresponding chromosome and physical position in the Bd21 reference genome (version 3).
(PDF)

S4 Fig Linkage groups of Luc1 x Jer1 genetic map. Cumulative cM distances and SNP marker names are shown to the left and right of each chromosome, respectively. cM distance at the F_2_ stage was estimated using the Kosambi function. SNP marker names consist of the corresponding chromosome and physical position in the Bd21 reference genome (version 3).
(PDF)

S5 Fig Two-way recombination fraction plots for the Foz1 x Luc1 F_2_ population (A) and the Luc1 x Jer1 F_2_ population (B). (PDF)

S6 Fig Segregation distortion in the Foz1 x Luc1 (A) and the Luc1 x Jer1 (B) F_2_ populations. For each marker of the genetic maps, the frequencies of F_2_ individuals with homozygous maternal genotypes (solid magenta lines), homozygous paternal genotypes (dashed green lines), or heterozygous genotypes (solid black lines) were calculated (scale on left). Data coverage (percentage of F_2_ individuals with genotype calls per marker) is represented by the gray lines (scale on right).
(PDF)

S7 Fig Composite interval mapping of leaf browning (orange) and pCOL (purple) in response to the four *P*. *striiformis* isolates based on individual replicates in the ABR6 x Bd21 F_4:5_ families. Phenotypes of F_4:5_ families were scored at 14 dpi with *P*. *striiformis* f. sp. *tritici* (*Pst*) isolates 08/21 (A and B), 08/501 (C and D), and 11/08 (E and F), and *P*. *striiformis* f. sp. *hordei* (*Psh*) isolate B01/2 (G and H). Composite interval mapping was performed under an additive model (H_0_:H_1_). Results were plotted based on normalized permutation thresholds (nLOD), using the threshold of statistical significance based on 1,000 permutations (blue horizontal line). R1 = replicate 1; R2 = replicate 2.
(PDF)

S8 Fig Composite interval mapping of leaf browning and pCOL in response to *P*. *striiformis* f. sp. *tritici* isolate 08/21 in the Foz1 x Luc1 (A) and Luc1 x Jer1 (B) F_2_ populations. F_2_ lines were phenotyped for leaf browning at 14 dpi (magenta) and at 23 dpi (yellow), for pCOL at 23 dpi (green), and Luc1 x Jer1 F_2:3_ families were phenotyped at 14 dpi (orange). Composite interval mapping was performed under an additive and dominance model (H_0_:H_3_). Results were plotted based on normalized permutation thresholds (nLOD), using the threshold of statistical significance based on 1,000 permutations (blue horizontal line).
(PDF)

S9 Fig Phylogenetic tree of *P*. *striiformis* isolates using maximum likelihood. DNA or RNA indicate genome or transcriptome sequencing. Tree branches represent nucleotide substitution rates (per million sites) and bootstrap values above 70 (based on 1,000 replicates) are shown.
(PDF)

S1 Table QTLs from composite interval mapping of individual replicates in the ABR6 x Bd21 F_4:5_ population. (PDF)

S2 Table Two cleaved amplified polymorphic sequences (CAPS) markers used to genotype Foz1 x Luc1 and Luc1 x Jer1 F_1_ plants. Markers are adapted from Barbieri *et al*. 2012. (PDF)

S1 File Significant QTLs from two-dimensional QTL analysis using leaf browning and percent colonization phenotypes for diverse *P*. *striiformis* isolates and *B*. *distachyon* populations. (XLSX)

S2 File Support intervals determined by interval mapping of the pCOL phenotypes and corresponding physical positions in the Bd21 reference genome. (XLSX)

S3 File Candidate gene analysis in *Yrr1* maximal two-LOD support interval. (XLSX)

S4 File Candidate gene analysis in *Yrr3* maximal two-LOD support interval. (XLSX)

S5 File Gene identifiers for the annotated canonical resistance genes shown in Fig 5. (XLSX)

S6 File Structural variation and gene expression information for the annotated canonical resistance genes shown in Fig 5. (XLSX)

S7 File KASP primers for the Foz1 x Luc1 and Luc1 x Jer1 genetic maps. (XLSX)

S8 File Foz1 x Luc1 genetic map. (XLSX)

S9 File Luc1 x Jer1 genetic map. (XLSX)

S10 File Leaf browning and pCOL phenotype data for the ABR6 x Bd21 F_4:5_ families. (XLSX)

S11 File Leaf browning and pCOL phenotype data for the Foz1 x Luc1 and Luc1 x Jer1 F_2_ populations. (XLSX)

S12 File Coding sequences of annotated *B*. *distachyon* reference genes in the *Yrr1* and *Yrr3* intervals converted into the ABR6, Luc1, and Jer1 genotypes. (FA)
