# Supplementary figures and images for: The genetic architecture of colonization resistance in Brachypodium distachyon to non-adapted stripe rust (Puccinia striiformis) isolates
Source: PLoS Genet. 2018 Sep 28;14(9):e1007637. doi: 10.1371/journal.pgen.1007637 (PMC6161849; doi:10.1371/journal.pgen.1007637)

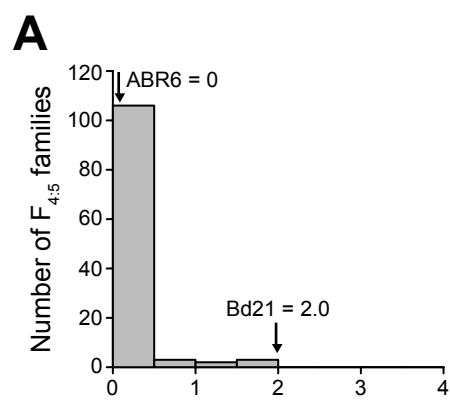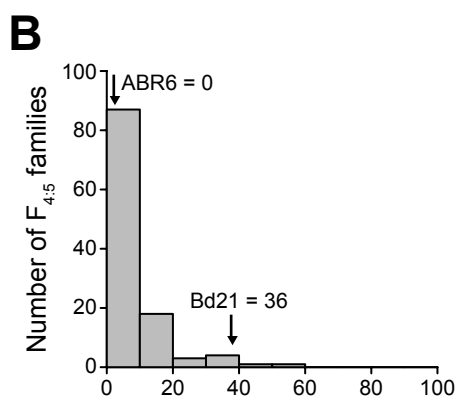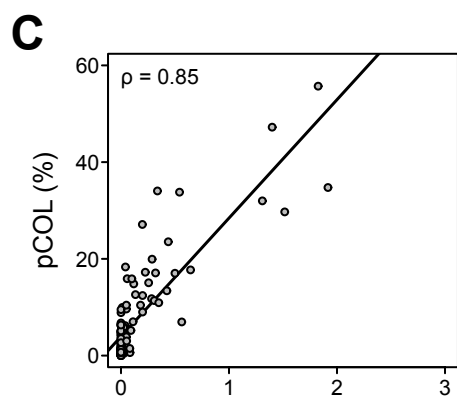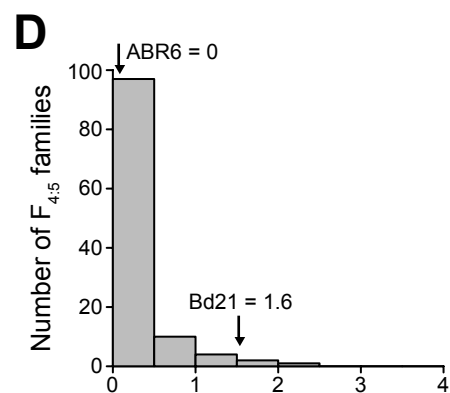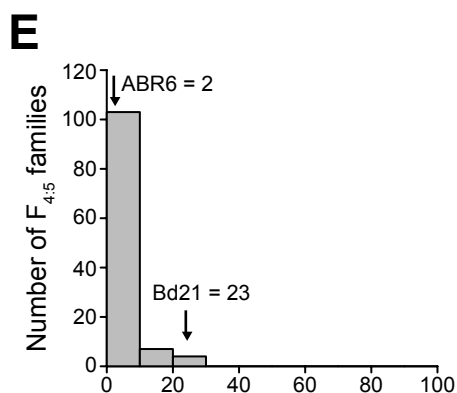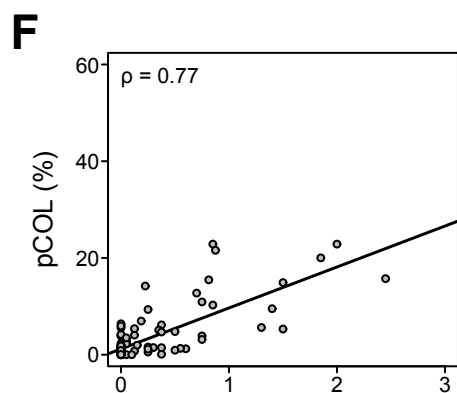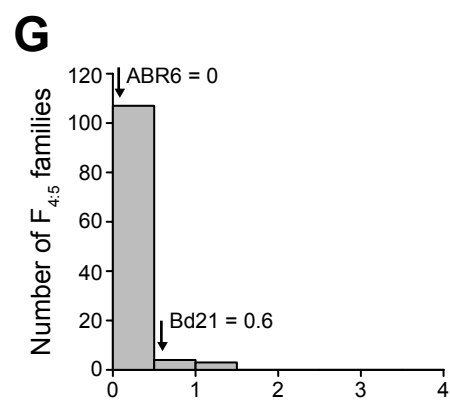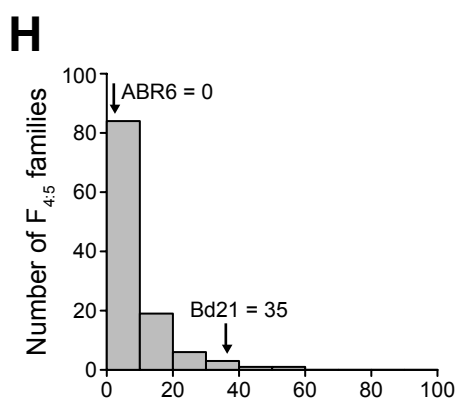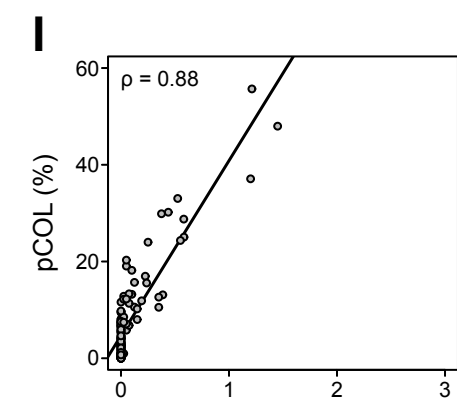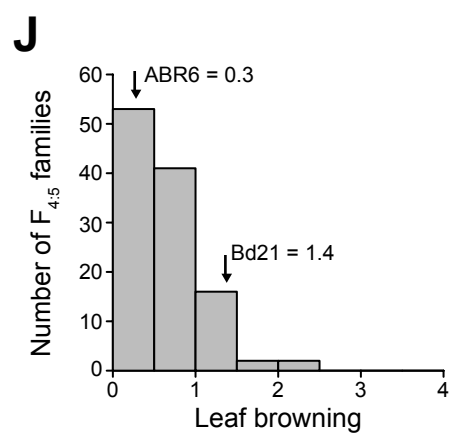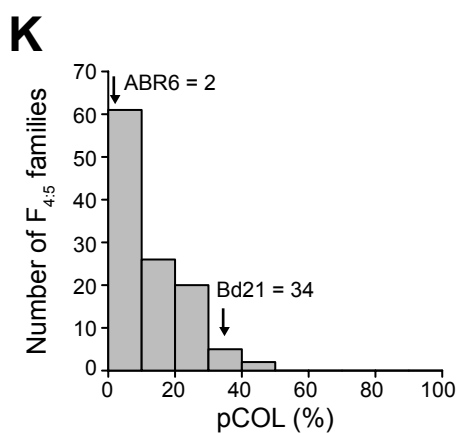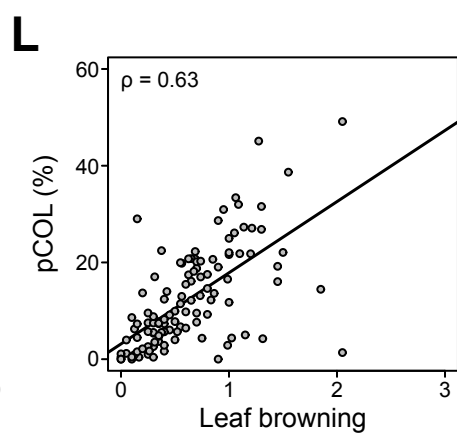

Supplement: S1 Fig — Distribution of leaf browning (A, D, G, and J) and pCOL (B, E, H, and K) and the correlation between these two phenotypes (C, F, I, and L) in the F4:5 families averaged across the two replicates for Pst isolates 08/21 (A–C), 08/501 (D–F), and 11/08 (G–I), and for Psh isolate B01/2 (J–L). Arrows indicate parental phenotypes. ρ = correlation coefficient. (PDF) [file pgen.1007637.s001.pdf]

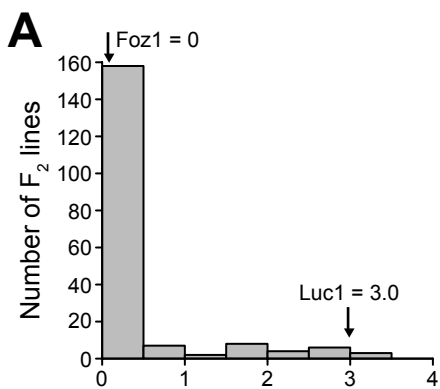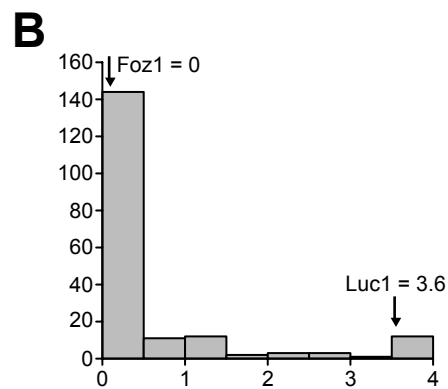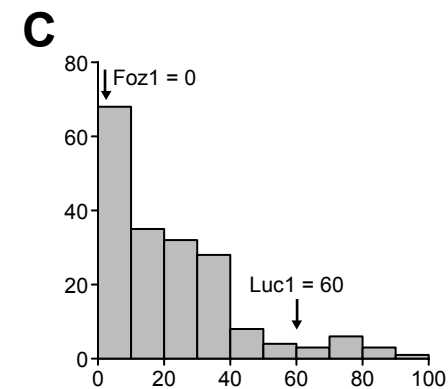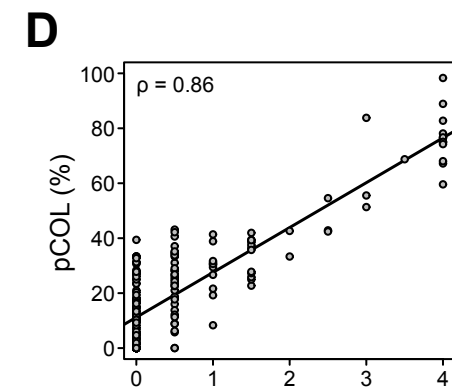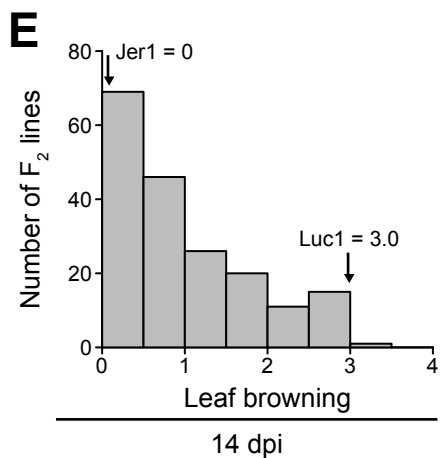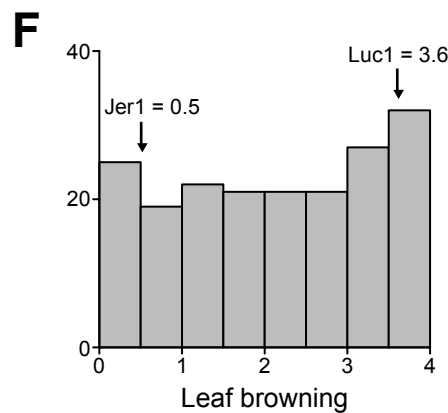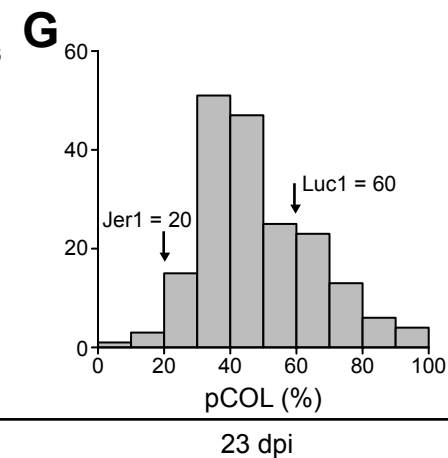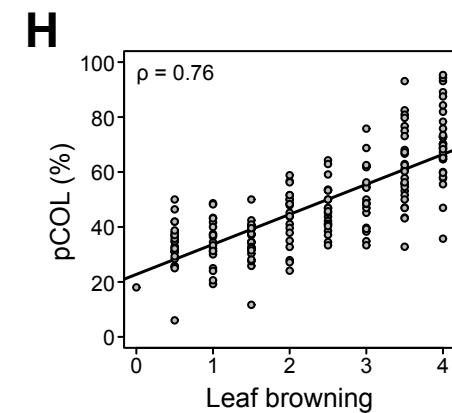

Supplement: S2 Fig — Leaf browning phenotypes were collected at 14 dpi (A and E) and at 23 dpi (B and F), and pCOL phenotypes were collected at 23 dpi (C and G). Correlation between leaf browning and pCOL phenotypes at 23 dpi is shown (D and H). Arrows indicate parental phenotypes. dpi = days post inoculation; ρ = correlation coefficient. (PDF) [file pgen.1007637.s002.pdf]

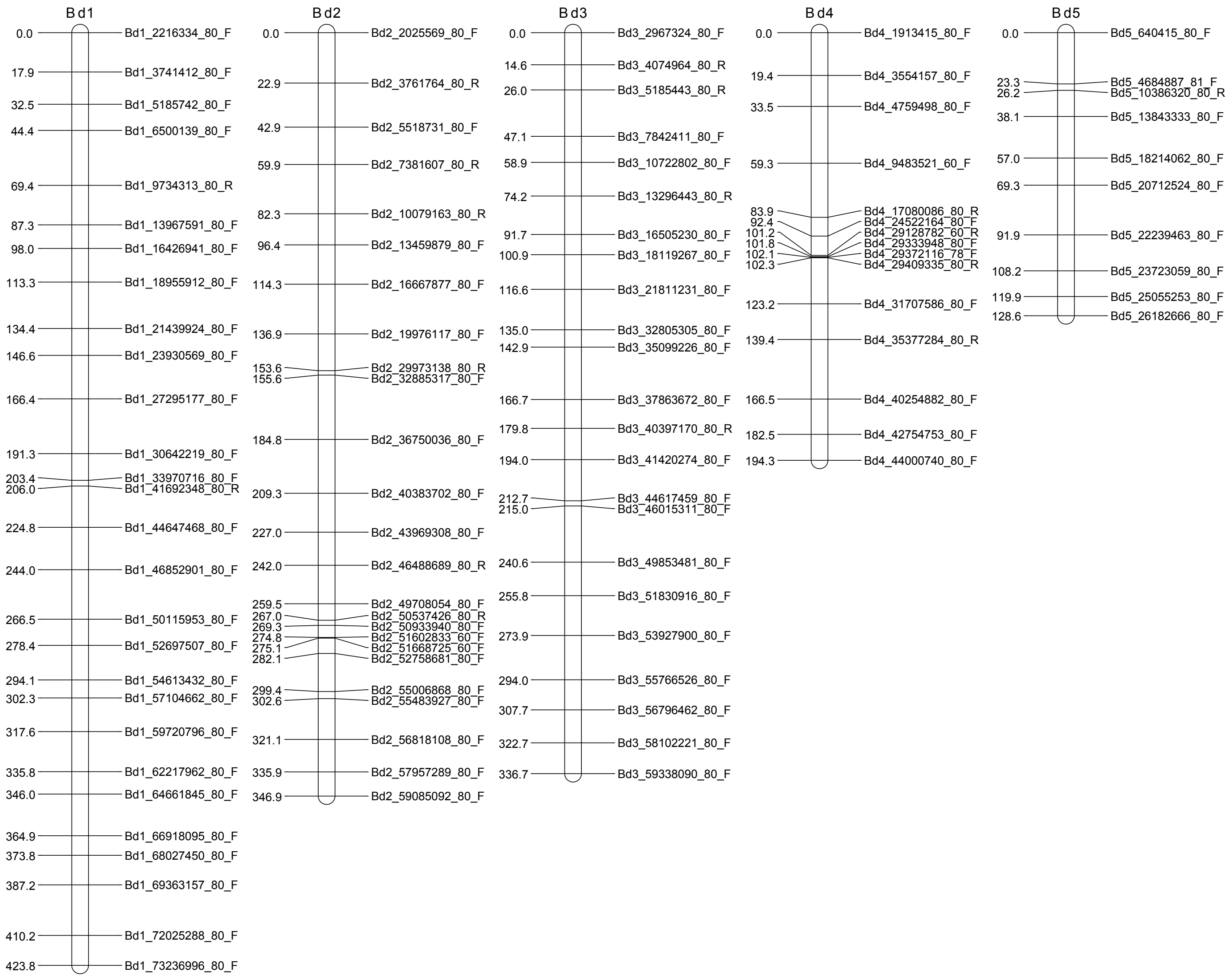

Supplement: S3 Fig — Cumulative cM distances and SNP marker names are shown to the left and right of each chromosome, respectively. cM distance at the F2 stage was estimated using the Kosambi function. SNP marker names consist of the corresponding chromosome and physical position in the Bd21 reference genome (version 3). (PDF) [file pgen.1007637.s003.pdf]

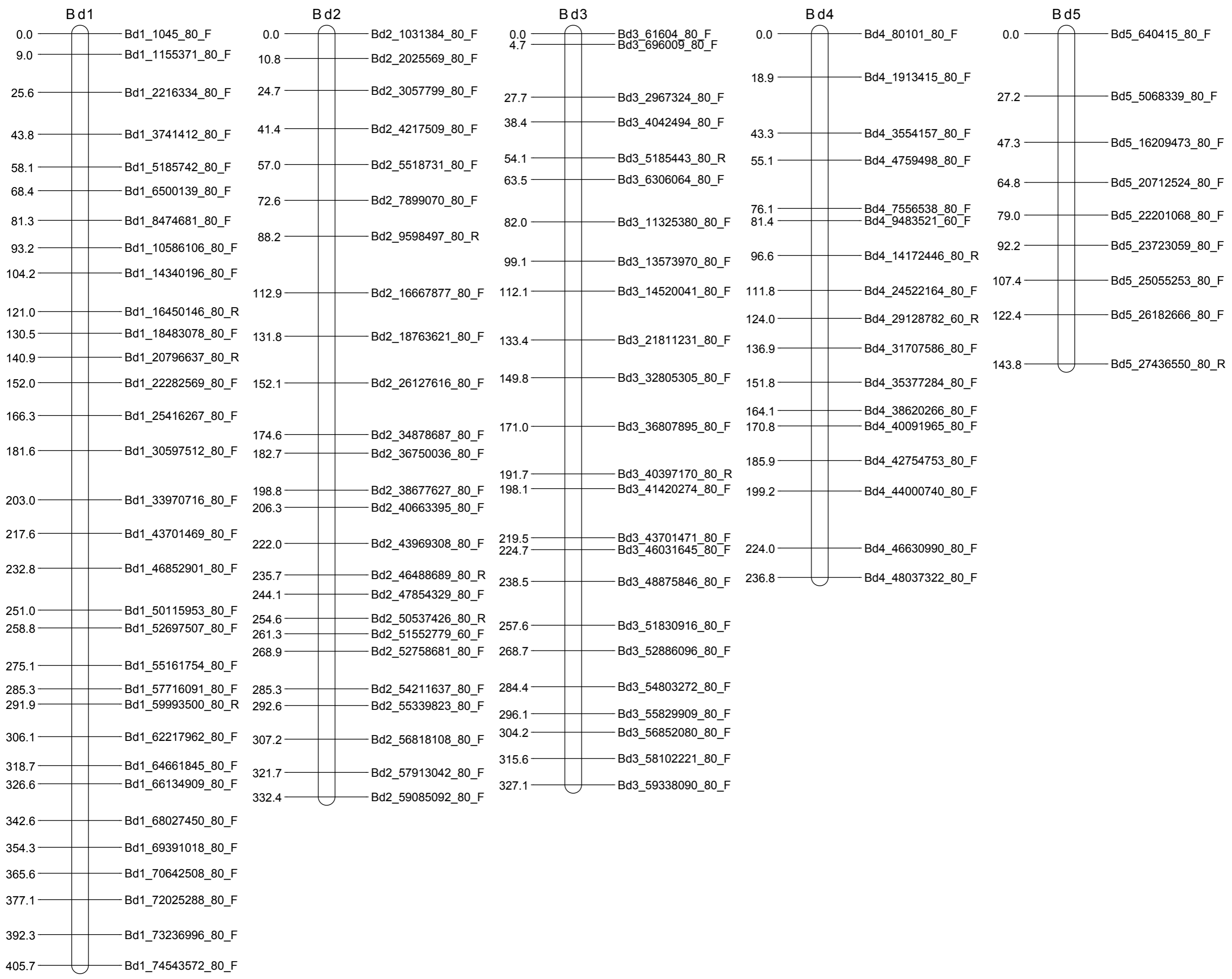

Supplement: S4 Fig — Cumulative cM distances and SNP marker names are shown to the left and right of each chromosome, respectively. cM distance at the F2 stage was estimated using the Kosambi function. SNP marker names consist of the corresponding chromosome and physical position in the Bd21 reference genome (version 3). (PDF) [file pgen.1007637.s004.pdf]

**A**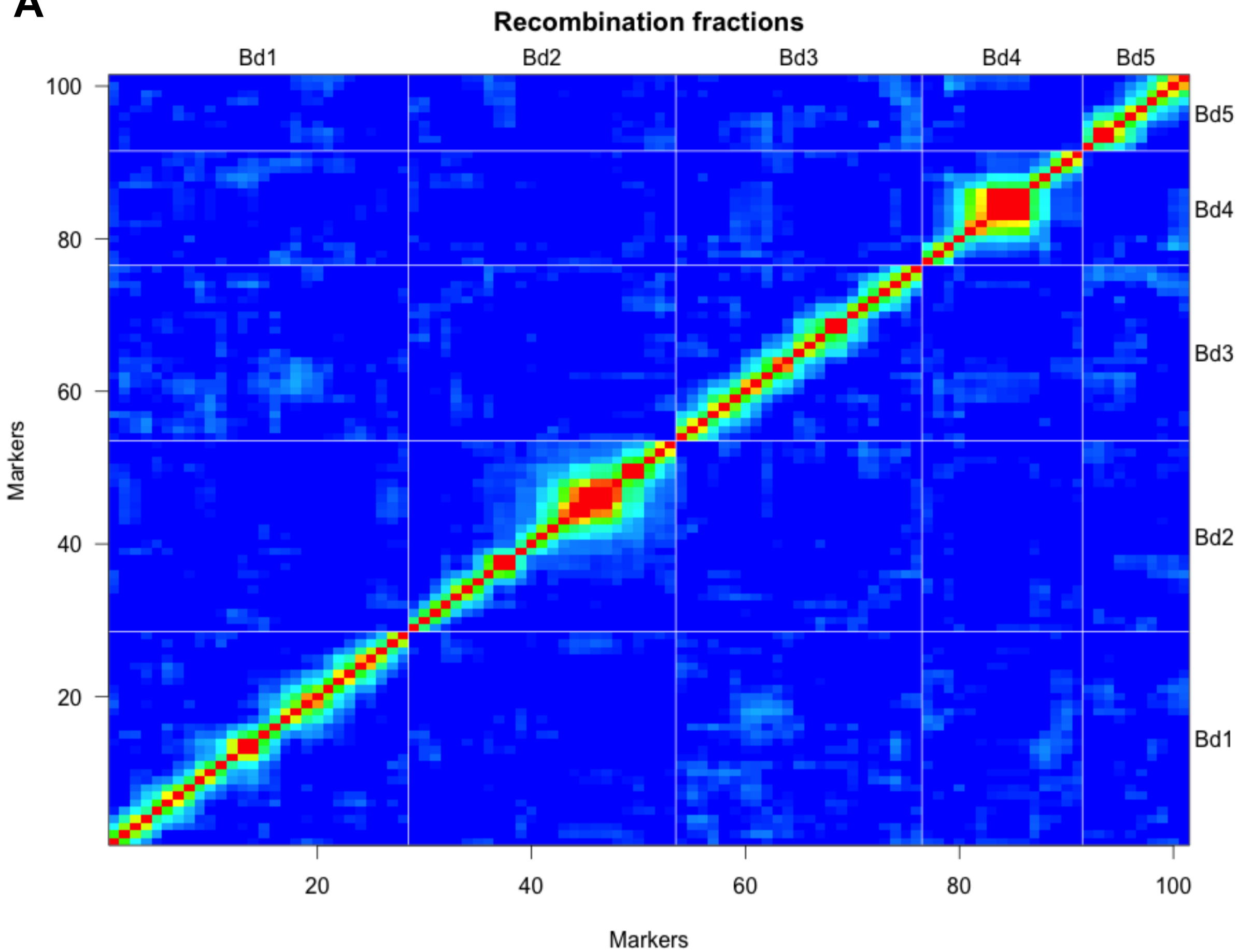**B**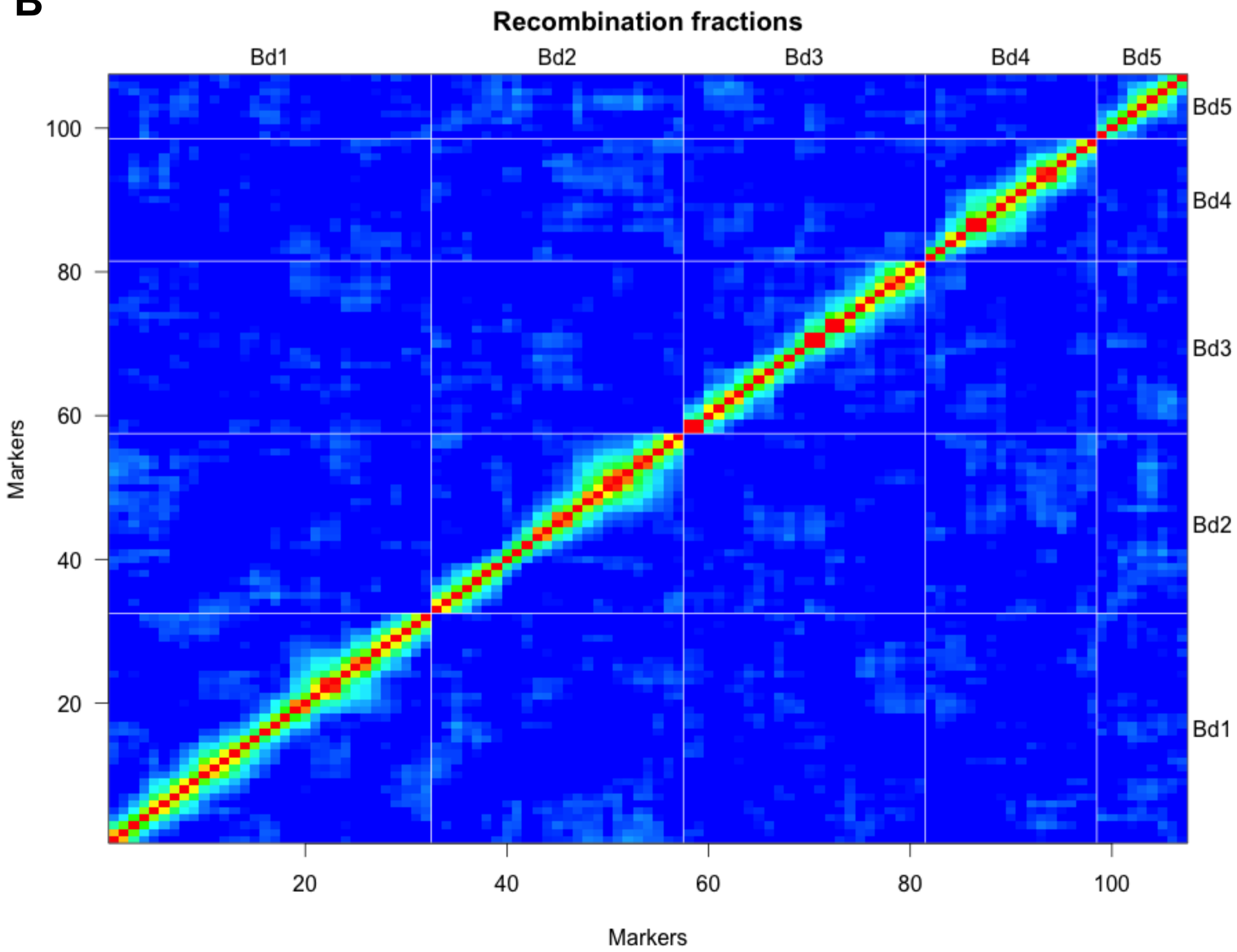

Supplement: S5 Fig — (PDF) [file pgen.1007637.s005.pdf]

**A**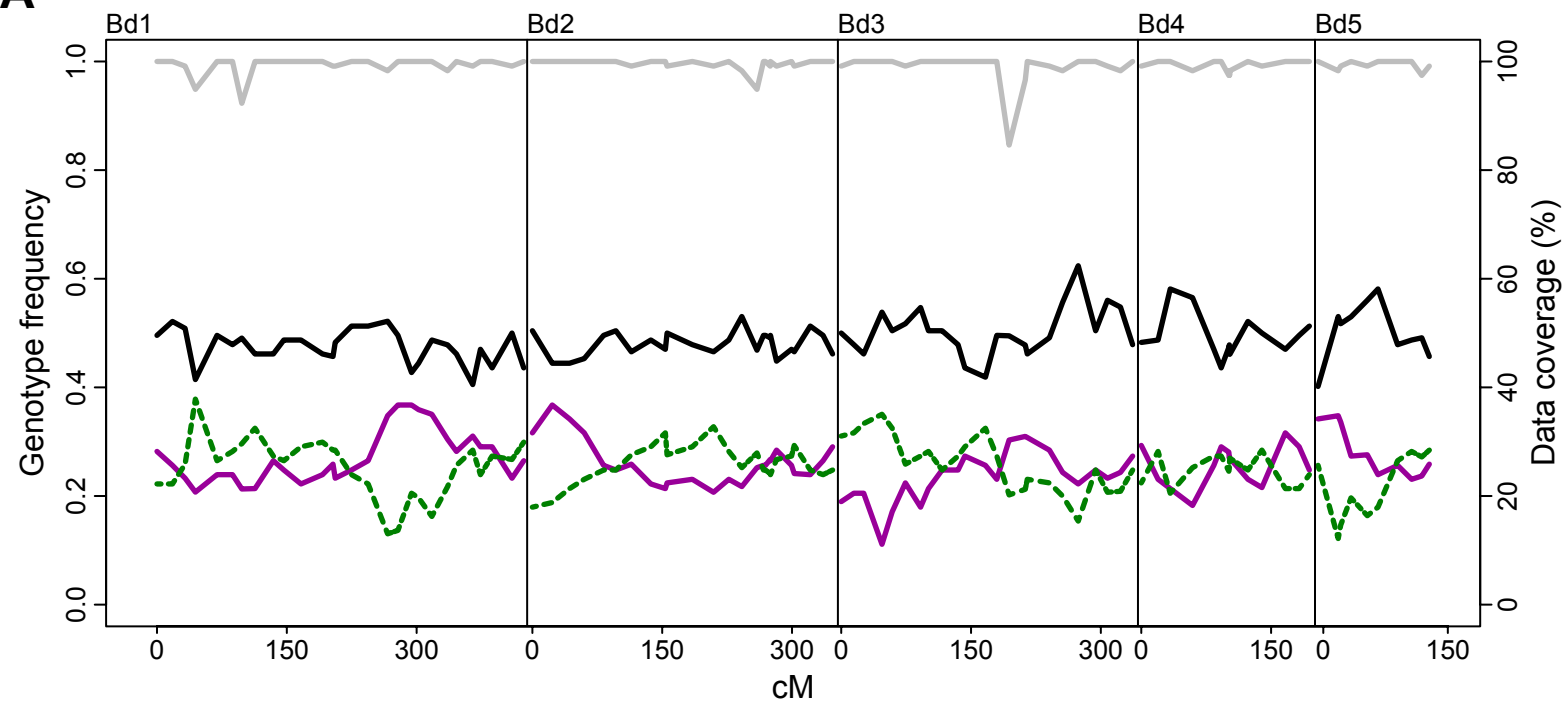**B**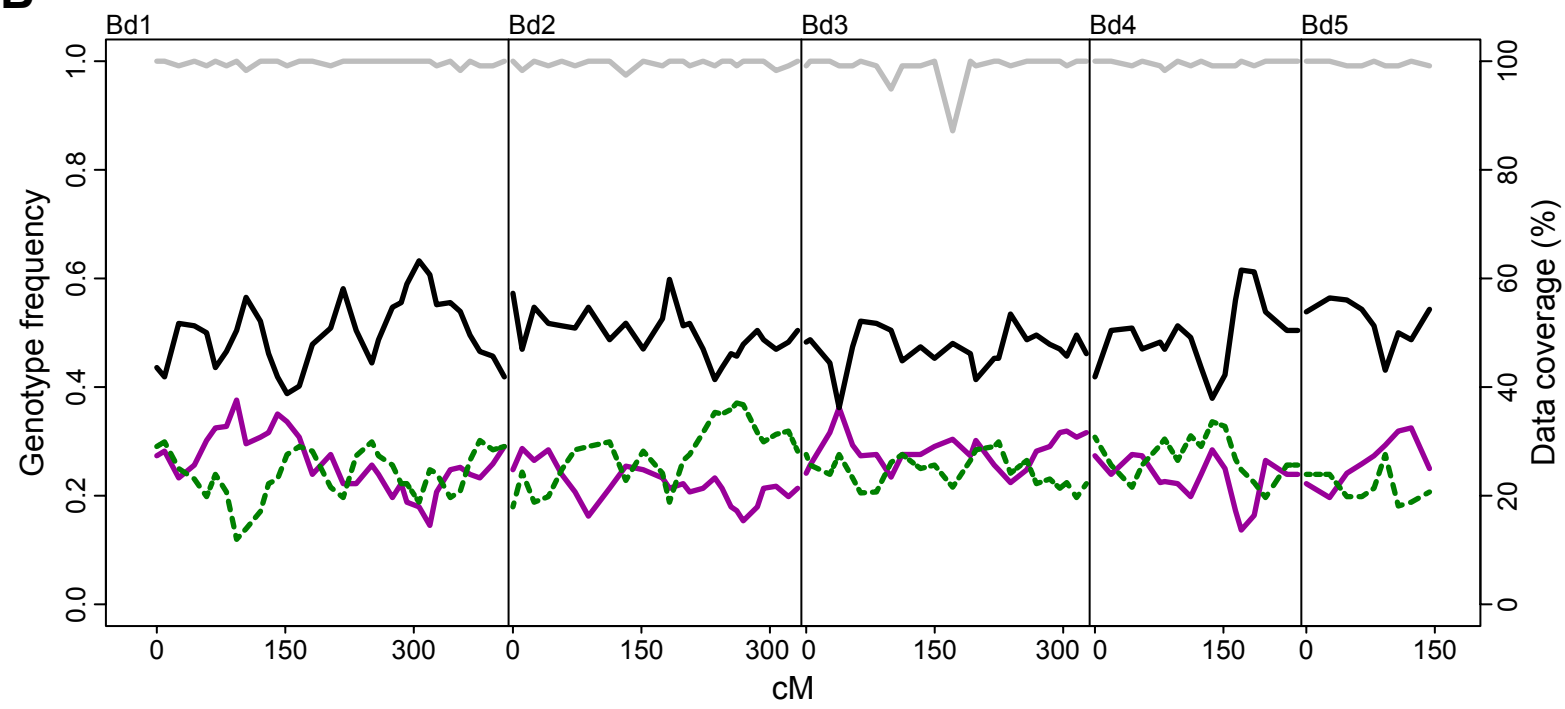

Supplement: S6 Fig — For each marker of the genetic maps, the frequencies of F2 individuals with homozygous maternal genotypes (solid magenta lines), homozygous paternal genotypes (dashed green lines), or heterozygous genotypes (solid black lines) were calculated (scale on left). Data coverage (percentage of F2 individuals with genotype calls per marker) is represented by the gray lines (scale on right). (PDF) [file pgen.1007637.s006.pdf]

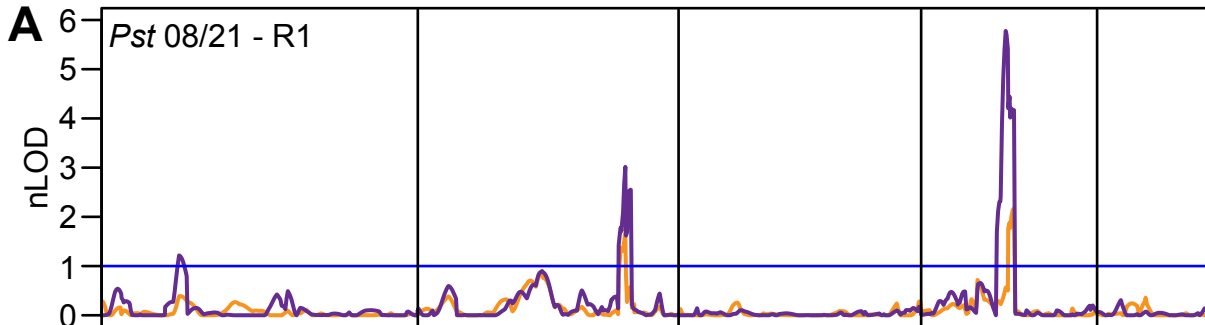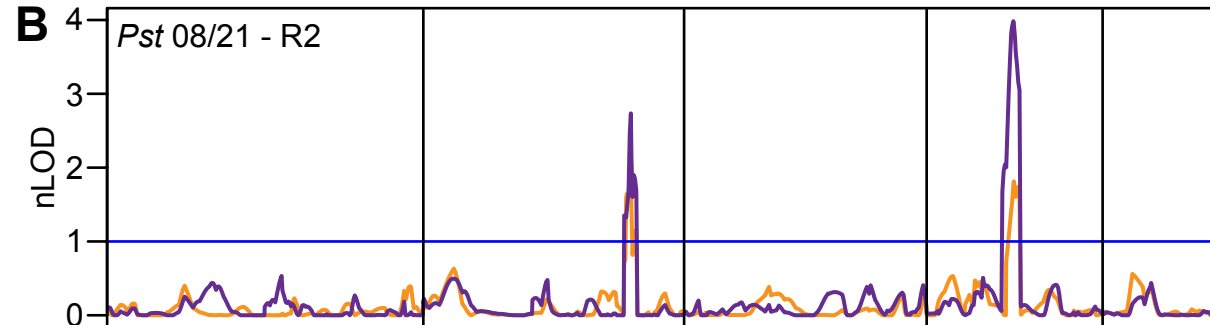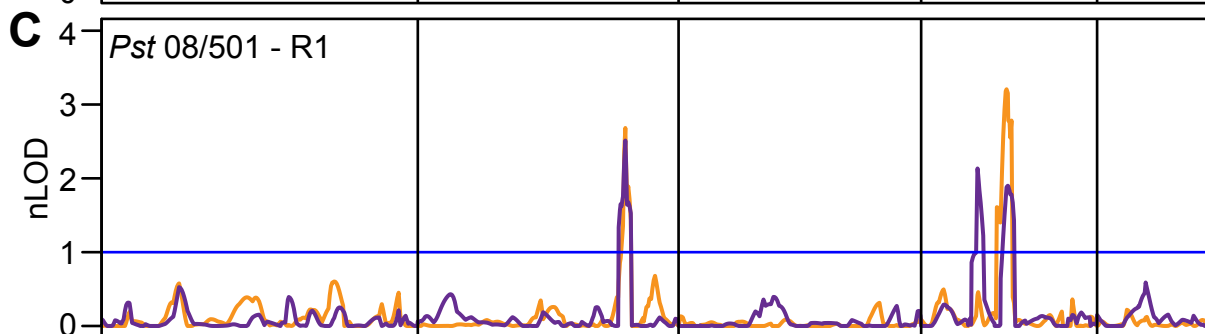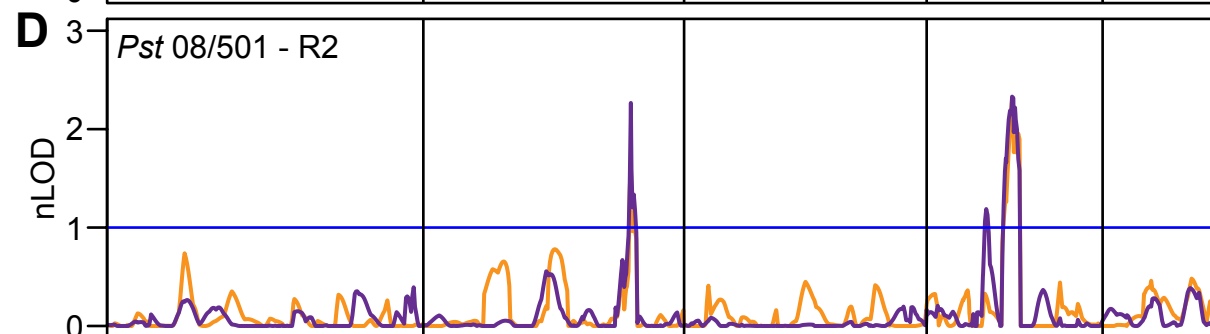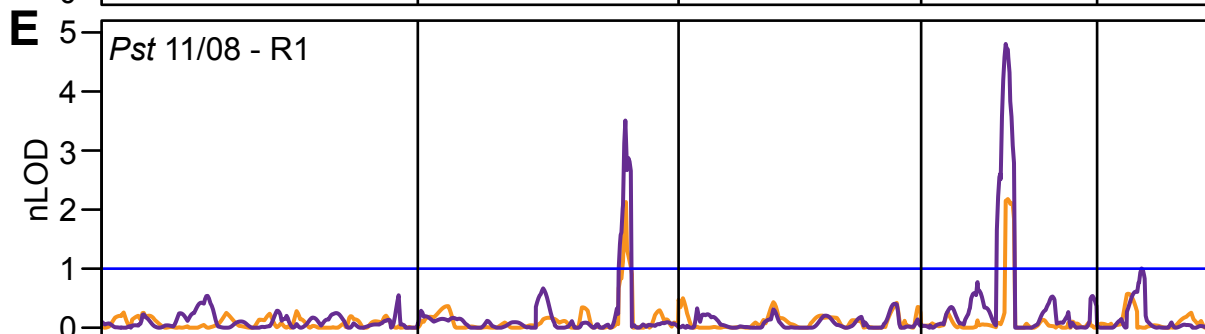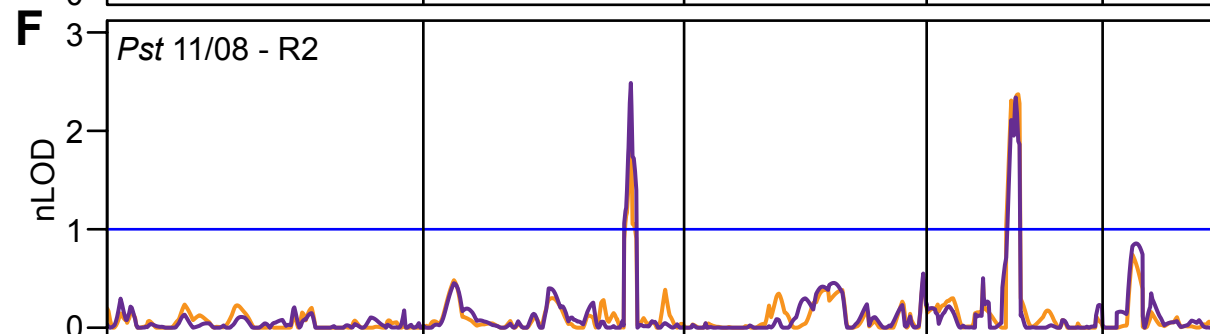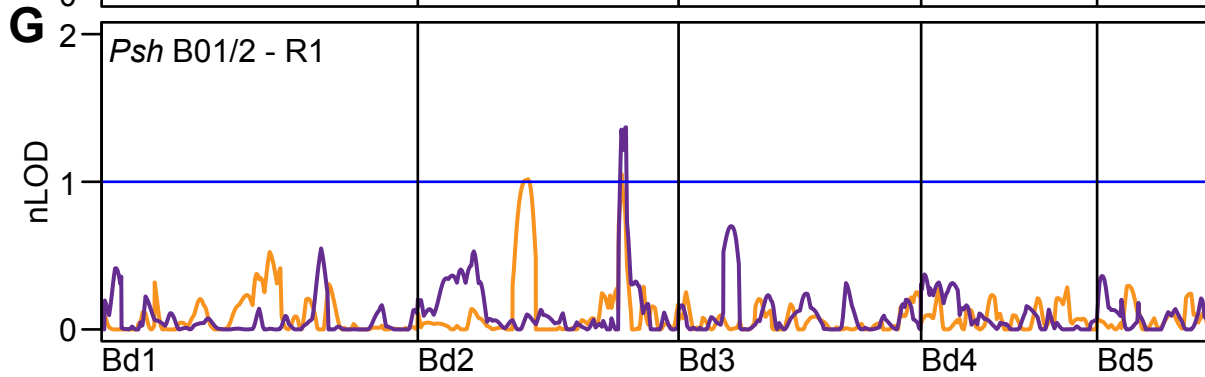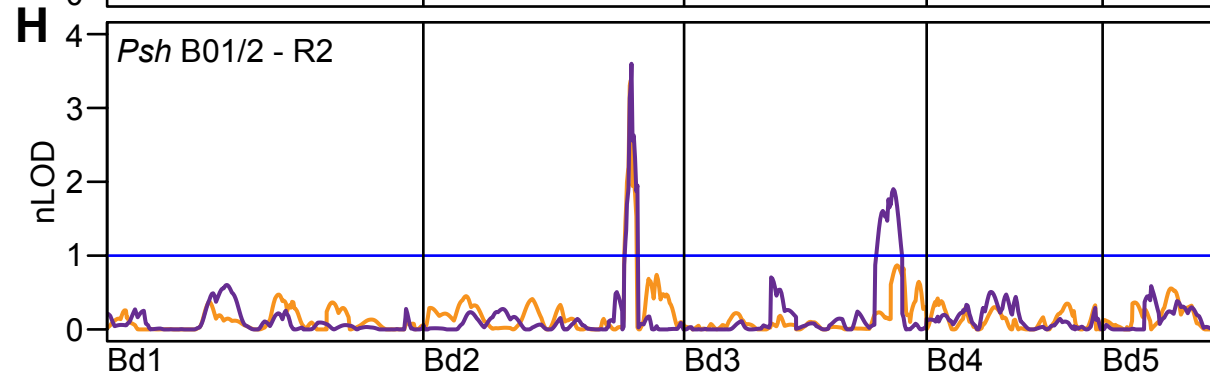

Supplement: S7 Fig — Phenotypes of F4:5 families were scored at 14 dpi with P. striiformis f. sp. tritici (Pst) isolates 08/21 (A and B), 08/501 (C and D), and 11/08 (E and F), and P. striiformis f. sp. hordei (Psh) isolate B01/2 (G and H). Composite interval mapping was performed under an additive model (H0:H1). Results were plotted based on normalized permutation thresholds (nLOD), using the threshold of statistical significance based on 1,000 permutations (blue horizontal line). R1 = replicate 1; R2 = replicate 2. (PDF) [file pgen.1007637.s007.pdf]

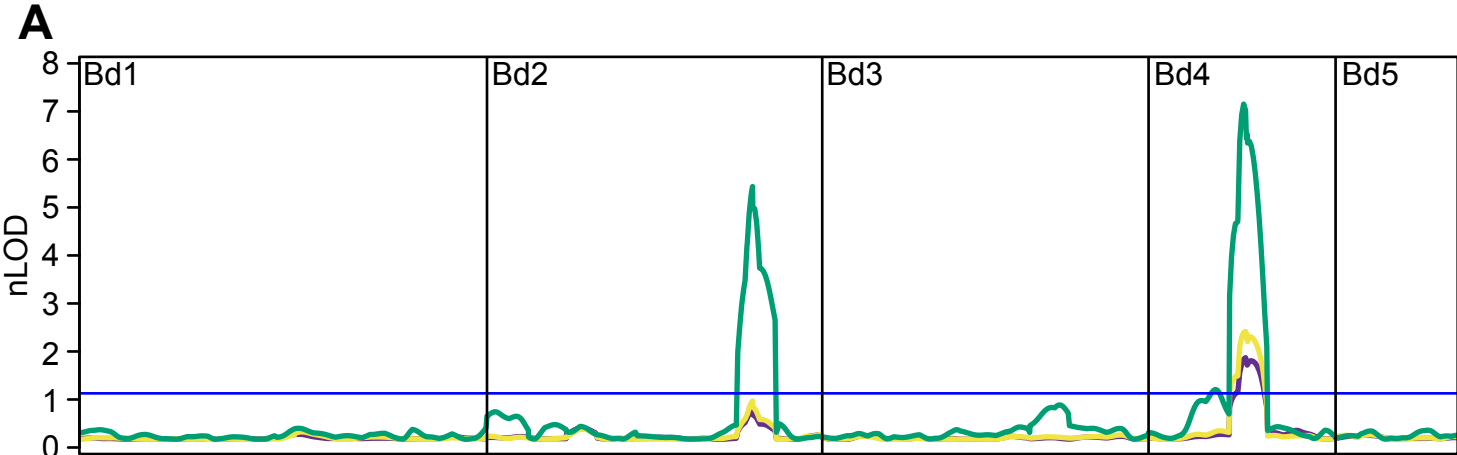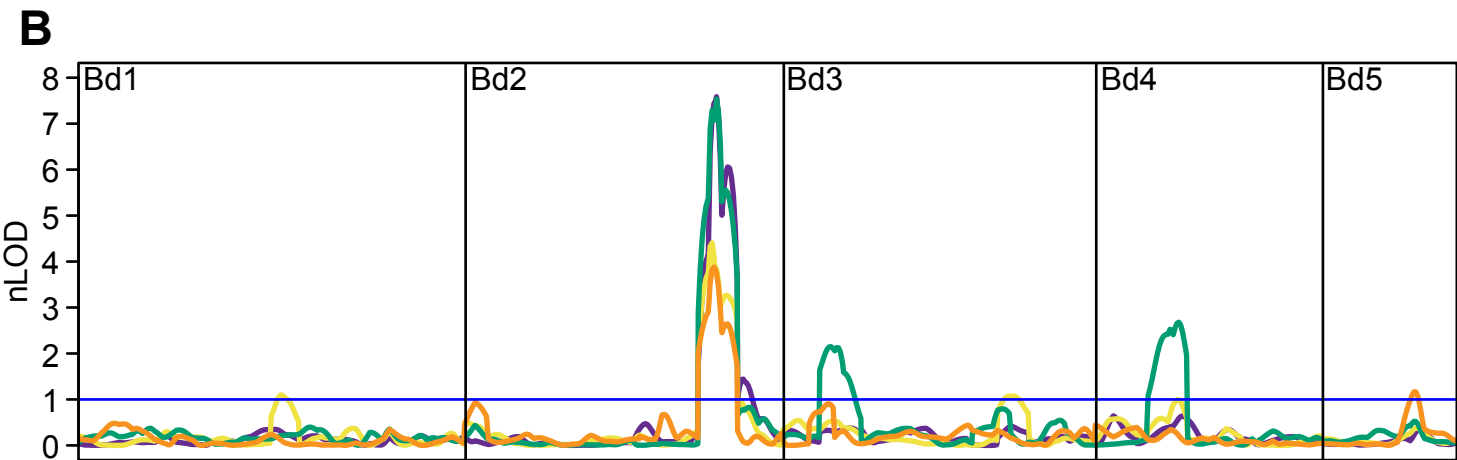

Supplement: S8 Fig — Composite interval mapping of leaf browning and pCOL in response to P. striiformis f. sp. tritici isolate 08/21 in the Foz1 x Luc1 (A) and Luc1 x Jer1 (B) F2 populations. F2 lines were phenotyped for leaf browning at 14 dpi (magenta) and at 23 dpi (yellow), for pCOL at 23 dpi (green), and Luc1 x Jer1 F2:3 families were phenotyped at 14 dpi (orange). Composite interval mapping was performed under an additive and dominance model (H0:H3). Results were plotted based on normalized permutation thresholds (nLOD), using the threshold of statistical significance based on 1,000 permutations (blue horizontal line). (PDF) [file pgen.1007637.s008.pdf]

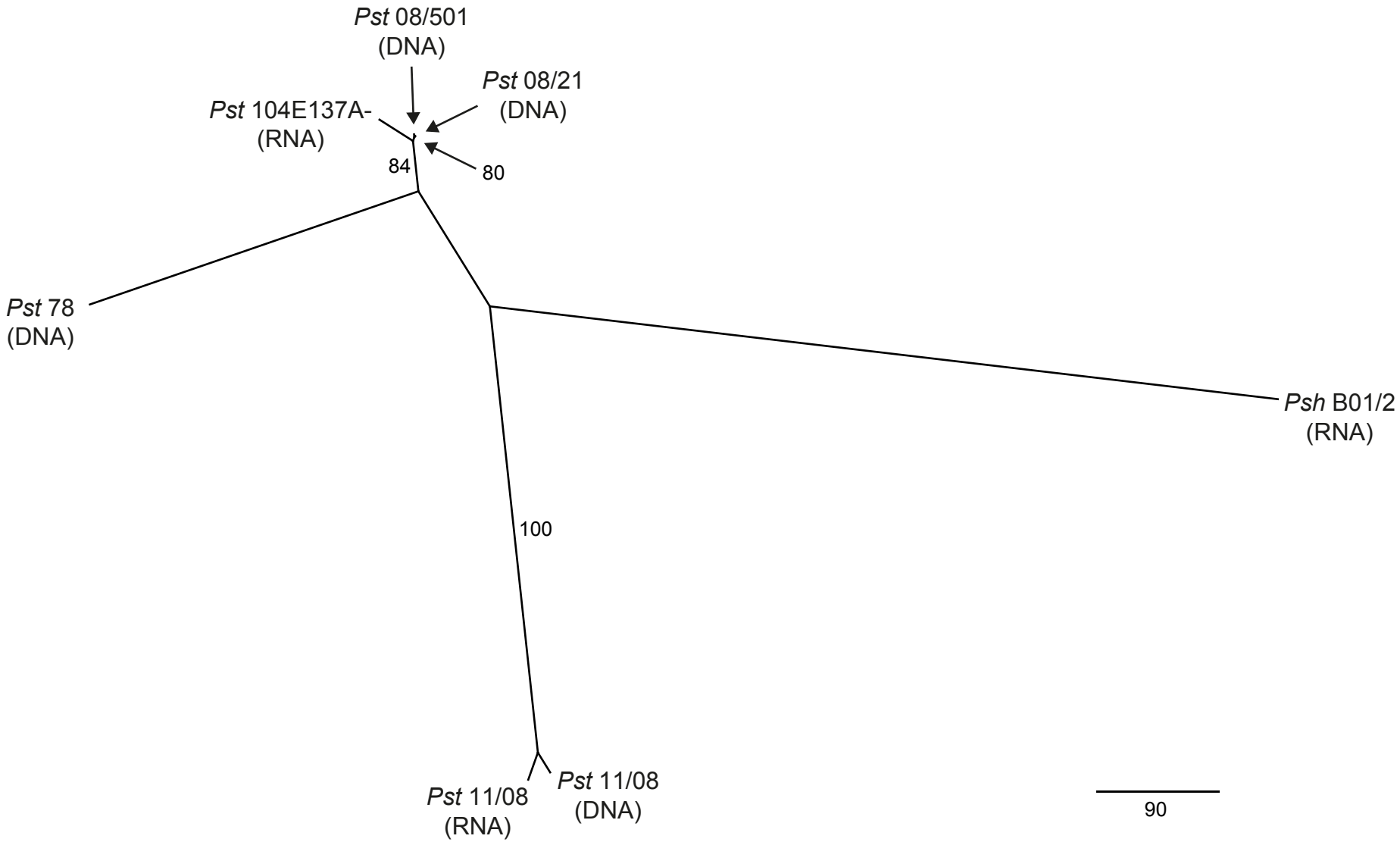

Supplement: S9 Fig — DNA or RNA indicate genome or transcriptome sequencing. Tree branches represent nucleotide substitution rates (per million sites) and bootstrap values above 70 (based on 1,000 replicates) are shown. (PDF) [file pgen.1007637.s009.pdf]
