# Supplementary material for: The genetic architecture of colonization resistance in Brachypodium distachyon to non-adapted stripe rust (Puccinia striiformis) isolates
Source: PLoS Genet. 2018 Sep 28;14(9):e1007637. doi: 10.1371/journal.pgen.1007637 (PMC6161849; doi:10.1371/journal.pgen.1007637)
Supplement: S1 Table — (PDF) [file pgen.1007637.s010.pdf]

**Table S1.** QTLs from composite interval mapping of individual replicates in the ABR6 x Bd21 $F_{4:5}$ population.

| Isolate[1] | Replicate | Trait[2] | Locus | Chr[3] | cM | EWT[4] | LOD | AEE[5] | PVE[6] |
|---|---|---|---|---|---|---|---|---|---|
| *Pst* 08/21 | 1 | Browning | *Yrr3* | Bd2 | 328.0 | 2.92 | 4.87 | -0.128 | 13.8 |
| *Pst* 08/21 | 1 | Browning | *Yrr1* | Bd4 | 146.8 | 2.92 | 6.35 | -0.124 | 13.1 |
| *Pst* 08/21 | 1 | pCOL | - | Bd1 | 124.3 | 2.86 | 3.48 | -0.026 | 4.5 |
| *Pst* 08/21 | 1 | pCOL | *Yrr3* | Bd2 | 328.0 | 2.86 | 8.62 | -0.051 | 18.2 |
| *Pst* 08/21 | 1 | pCOL | *Yrr1* | Bd4 | 133.6 | 2.86 | 16.52 | -0.068 | 32.5 |
| *Pst* 08/21 | 2 | Browning | *Yrr3* | Bd2 | 328.0 | 2.67 | 5.86 | -0.193 | 17.9 |
| *Pst* 08/21 | 2 | Browning | *Yrr1* | Bd4 | 137.7 | 2.67 | 4.85 | -0.162 | 11.4 |
| *Pst* 08/21 | 2 | pCOL | *Yrr3* | Bd2 | 328.0 | 2.83 | 7.74 | -0.047 | 18.8 |
| *Pst* 08/21 | 2 | pCOL | *Yrr1* | Bd4 | 137.0 | 2.83 | 11.28 | -0.057 | 26.5 |
| *Pst* 08/501 | 1 | Browning | *Yrr3* | Bd2 | 328.0 | 3.03 | 8.12 | -0.329 | 18.2 |
| *Pst* 08/501 | 1 | Browning | *Yrr1* | Bd4 | 135.0 | 3.03 | 9.71 | -0.374 | 23.5 |
| *Pst* 08/501 | 1 | pCOL | *Yrr3* | Bd2 | 328.0 | 2.74 | 6.89 | -0.027 | 17.1 |
| *Pst* 08/501 | 1 | pCOL | *Yrr2* | Bd4 | 89.2 | 2.74 | 5.85 | -0.025 | 12.1 |
| *Pst* 08/501 | 1 | pCOL | *Yrr1* | Bd4 | 137.0 | 2.74 | 5.21 | -0.021 | 9.9 |
| *Pst* 08/501 | 2 | Browning | *Yrr3* | Bd2 | 328.0 | 2.69 | 4.52 | -0.117 | 13.9 |
| *Pst* 08/501 | 2 | Browning | *Yrr1* | Bd4 | 135.0 | 2.69 | 6.22 | -0.131 | 17.7 |
| *Pst* 08/501 | 2 | pCOL | *Yrr3* | Bd2 | 328.0 | 3.06 | 6.94 | -0.030 | 18.0 |
| *Pst* 08/501 | 2 | pCOL | *Yrr2* | Bd4 | 94.1 | 3.06 | 3.64 | -0.021 | 11.7 |
| *Pst* 08/501 | 2 | pCOL | *Yrr1* | Bd4 | 135.0 | 3.06 | 7.13 | -0.024 | 15.4 |
| *Pst* 11/08 | 1 | Browning | *Yrr3* | Bd2 | 328.9 | 2.66 | 5.65 | -0.066 | 14.4 |
| *Pst* 11/08 | 1 | Browning | *Yrr1* | Bd4 | 137.0 | 2.66 | 5.79 | -0.066 | 13.9 |
| *Pst* 11/08 | 1 | pCOL | *Yrr3* | Bd2 | 328.0 | 3.15 | 11.06 | -0.049 | 18.9 |
| *Pst* 11/08 | 1 | pCOL | *Yrr2* | Bd4 | 133.6 | 3.15 | 15.15 | -0.065 | 33.8 |
| *Pst* 11/08 | 1 | pCOL | - | Bd5 | 70.3 | 3.15 | 3.17 | -0.029 | 6.3 |
| *Pst* 11/08 | 2 | Browning | *Yrr3* | Bd2 | 328.0 | 2.59 | 5.07 | -0.137 | 13.1 |
| *Pst* 11/08 | 2 | Browning | *Yrr1* | Bd4 | 144.8 | 2.59 | 6.15 | -0.143 | 14.2 |
| *Pst* 11/08 | 2 | pCOL | *Yrr3* | Bd2 | 328.0 | 2.77 | 6.88 | -0.049 | 17.3 |
| *Pst* 11/08 | 2 | pCOL | *Yrr1* | Bd4 | 140.8 | 2.77 | 6.47 | -0.044 | 12.8 |
| *Psh* B01/2 | 1 | Browning | - | Bd2 | 174.4 | 3.01 | 3.07 | 0.168 | 13.7 |
| *Psh* B01/2 | 1 | Browning | *Yrr3* | Bd2 | 321.7 | 3.01 | 3.18 | -0.136 | 9.4 |
| *Psh* B01/2 | 1 | pCOL | *Yrr3* | Bd2 | 328.9 | 2.99 | 4.10 | -0.035 | 18.7 |
| *Psh* B01/2 | 2 | Browning | *Yrr3* | Bd2 | 328.0 | 3.15 | 10.64 | -0.364 | 28.9 |
| *Psh* B01/2 | 2 | pCOL | *Yrr3* | Bd2 | 328.9 | 3.15 | 11.35 | -0.098 | 26.1 |
| *Psh* B01/2 | 2 | pCOL | - | Bd3 | 330.6 | 3.15 | 5.99 | -0.063 | 12.4 |

[1] *Puccinia striiformis* isolate (*Pst* = f. sp. *tritici*, *Psh* = f. sp. *hordei*)
[2] Browning = leaf browning; pCOL = percent colonization
[3] Chromosome
[4] Experiment-wide permutation threshold
[5] Additive effect estimate
[6] Percent of variation explained of variation explained
