# Supplementary material for: The genetic architecture of colonization resistance in Brachypodium distachyon to non-adapted stripe rust (Puccinia striiformis) isolates
Source: PLoS Genet. 2018 Sep 28;14(9):e1007637. doi: 10.1371/journal.pgen.1007637 (PMC6161849; doi:10.1371/journal.pgen.1007637)
Supplement: S2 Table — (PDF) [file pgen.1007637.s011.pdf]

Table S2. Two cleaved amplified polymorphic sequences (CAPS) markers used to genotype Foz1 x Luc1 and Luc1 x Jer1 F_1 plants. Markers are adapted from Barbieri *et al.* 2012.

Cross	Marker	Primers (forward; reverse)	Temp.[1]	Restriction enzyme
Foz1 x Luc1	Bradi4g10930	GGTTGAGTTATTGCCGTCAG; CGCCTTTGGATCTACCAGTC	56˚C	MseI
Luc1 x Jer1	Bradi4g10930	GGTTGAGTTATTGCCGTCAG; CGCCTTTGGATCTACCAGTC	56˚C	MboI

[1]Annealing temperature
